# Alternative Splicing and Subfunctionalization Generates Functional Diversity in Fungal Proteomes

**DOI:** 10.1371/journal.pgen.1003376

**Published:** 2013-03-14

**Authors:** Alexandra N. Marshall, Maria Camila Montealegre, Claudia Jiménez-López, Michael C. Lorenz, Ambro van Hoof

**Affiliations:** Department of Microbiology and Molecular Genetics, University of Texas Health Science Center at Houston, Houston, Texas, United States of America; Duke University Medical Center, United States of America

## Abstract

Alternative splicing is commonly used by the *Metazoa* to generate more than one protein from a gene. However, such diversification of the proteome by alternative splicing is much rarer in fungi. We describe here an ancient fungal alternative splicing event in which these two proteins are generated from a single alternatively spliced ancestral *SKI7/HBS1* gene retained in many species in both the Ascomycota and Basidiomycota. While the ability to express two proteins from a single *SKI7/HBS1* gene is conserved in many fungi, the exact mechanism by which they achieve this varies. The alternative splicing was lost in *Saccharomyces cerevisiae* following the whole-genome duplication event as these two genes subfunctionalized into the present functionally distinct *HBS1* and *SKI7* genes. When expressed in yeast, the single gene from *Lachancea kluyveri* generates two functionally distinct proteins. Expression of one of these proteins complements hbs1, but not ski7 mutations, while the other protein complements ski7, but not hbs1. This is the first known case of subfunctionalization by loss of alternative splicing in yeast. By coincidence, the ancestral alternatively spliced gene was also duplicated in *Schizosaccharomyces pombe* with subsequent subfunctionalization and loss of splicing. Similar subfunctionalization by loss of alternative splicing in fungi also explains the presence of two *PTC7* genes in the budding yeast *Tetrapisispora blattae*, suggesting that this is a common mechanism to preserve duplicate alternatively spliced genes.

## Author Summary

The role of duplicated genes in originating new functions is an important question in evolution. Almost all species have duplicated genes that carry out similar but not identical functions. Similar proteins that perform different functions can also be generated when one gene generates multiple mRNAs by alternative splicing that are translated into multiple similar proteins. This alternative splicing is prevalent in animal cells, but much rarer in fungi. Here we show that most fungi use alternative splicing to make a Ski7 protein and a Hbs1 protein from the same gene. Two fungi, budding yeast and fission yeast, have been much better characterized than other fungi, and co-incidentally they both have duplicated this alternatively spliced gene, resulting in two similar genes that are no longer alternatively spliced. Finally, we describe another example where two duplicate genes replace one alternatively spliced gene, suggesting that this is a common mechanism to divide functions among duplicate genes.

## Introduction

Gene duplication is thought to be a major source of evolutionary innovation. Although the fate of duplicated genes is incompletely understood, it is thought to fit one of three patterns: nonfunctionalization, neofunctionalization or subfunctionalization. Of these nonfunctionalization is thought to be the most common. Immediately after duplication, duplicated genes are typically redundant. Thus, purifying selection cannot provide selective pressure to maintain both. The absence of selective pressure generally leads to loss of function mutations (nonfunctionalization) in one of the copies, followed by loss of that copy of the gene. In neofunctionalization, one of the duplicated copies acquires a new advantageous function that is different from the ancestral function maintained by the other copy [1]. Subfunctionalization can occur when an ancestral gene carries out more than one function. If one duplicated copy mutates so that it loses one of the functions, and the other copy mutates so that it loses a separate function, selective pressure can subsequently maintain both copies by selecting for both functions [2], [3]. Multiple functions in this context can mean being expressed in multiple cell types, encoding proteins localized to different compartments, encoding proteins with distinct biochemical activities, etc.

*Saccharomyces cerevisiae* is an excellent model organism to study the fate of duplicated gene pairs because an ancestor underwent a whole genome duplication (WGD) approximately 100 million years ago resulting in a transient increase in genome size from around 5000 protein coding genes to 10,000 [4], [5], [6]. Following genome duplication most duplicated genes were lost (nonfunctionalized), but 544 duplicated gene pairs that arose from WGD remain [4]. The genomes of many related species have been sequenced, which revealed through synteny which genes were duplicated as part of the WGD [6], [7], [8]. The related genomes also provide a large amount of sequence information on the duplicated genes and their non-duplicated homologs. The pattern of gene retention in these genomes revealed that nonfunctionalization after WGD is random such that different post-WGD species retained different subsets of duplicated genes [9]. In addition, gene function can be easily assayed in *S. cerevisiae*. Using these advantages, we have previously shown that subfunctionalization is a major mechanism by which duplicated *S. cerevisiae* genes were retained, which was confirmed by others [10], [11], .

One example of subfunctionalized genes resulting from the WGD event is provided by *SKI7* and *HBS1* [11]. The *S. cerevisiae* Ski7 and Hbs1 proteins both recognize ribosomes stalled during translation and initiate degradation of the mRNA. However, they recognize different stalled ribosomes and initiate mRNA degradation differently. When an mRNA lacks an in frame stop codon, the ribosome is thought to translate until it reaches the 3′ end of that mRNA [13]. The stalled ribosome is then recognized by Ski7, which recruits the RNA exosome to degrade that mRNA. In contrast, Hbs1 recognizes ribosomes stalled within the coding region, for example due to a structure or damage in the mRNA [14]. Recognition by Hbs1 causes cleavage of the mRNA in an RNA exosome-independent manner [14], [15]. Although the *SKI7* and *HBS1* genes of *S. cerevisiae* perform distinct functions, we have previously shown that the single ancestral gene performed both functions [11]. The related budding yeast *Lachancea kluyveri* diverged from *S. cerevisiae* before WGD and thus contains a single ortholog to *SKI7* and *HBS1*, which we will call *SKI7/HBS1*. Our key finding was that when the *L. kluyveri SKI7/HBS1* gene was introduced into *S. cerevisiae* it could complement the defects caused by both *ski7Δ* and *hbs1Δ*, thus indicating that this single gene carried out both functions [11].

Since the function of duplicated genes can diverge from each other through neo- or subfunctionalization, gene duplication may be one way to generate a more diverse proteome. The proteome can also be diversified through alternative splicing, where one gene generates multiple distinct mRNAs that each encode a distinct protein. Although alternative splicing is important to diversify the proteome in metazoans, it is much rarer in the fungal kingdom. Most fungal alternative splicing events that have been described are of the intron retention type, where the spliced mRNA encodes a functional protein, and the unspliced mRNA is nonfunctional. For example, transcriptome sequencing of *Aspergillus oryzae* identified only 8.6% of the genes as alternatively spliced, which is 10-fold lower than in humans and 92% of the *Aspergillus* alternative splicing was intron retention [16]. A well-studied and typical example of fungal intron retention is the *S. cerevisiae CYH2* gene. The *CYH2* mRNA encodes a 17 KDa ribosomal protein. The intron in the *CYH2* pre-mRNA is retained approximately 50% of the time, which results in an mRNA that codes for a 2 KDa peptide with no known function. Furthermore, intron-retained mRNAs are typically very rapid degraded by the nonsense-mediated mRNA decay pathways [17]. In these cases instead of diversifying the proteome, intron retention may function to regulate gene expression. Similarly, the *S. cerevisiae SRC1* gene is alternatively spliced using alternative 5′ splice sites, but only the longer splice isoform has been shown to be functional [18], [19]. To the best of our knowledge, the only case in which intron retention or alternative splicing leads to two functional mRNAs in *S. cerevisiae* is in *PTC7*, which contains one intron and encodes a protein phosphatase subunit. If this intron is spliced out, the mRNA is translated into a protein that is imported into the mitochondria, while after intron retention the mRNA is translated into a protein that is inserted into the nuclear envelope [20]. A few other cases have been described were fungi use alternative splicing to target a protein to multiple locations [21], [22], [23].

As mentioned above, there are multiple ways a gene can be multifunctional in the context of subfunctionalization. A corollary of that is that subfunctionalization can occur through distinct molecular changes. In yeast, subfunctionalization through changes in the coding region seem to be common [10], [11], [12]. In these cases a single amino acid change can be responsible for subfunctionalization [10], [12]. In multicellular organisms, genes that are expressed in multiple cell types, in response to multiple stimuli, or by multiple transcription factors can be subfunctionalized through changes in expression pattern [e.g. ref 3]. Subfunctionalization through changes in splicing patterns have been described in a few cases [e.g. refs 24], [25]. In these cases, an alternatively spliced gene upon duplication results in two genes where one gene follows one ancestral splicing pattern and the other follows another ancestral splicing pattern. However, the function of these alternative splicing isoforms is often not clear. Thus while loss of alternative splicing happens at the same time as some gene duplications, whether they cause subfunctionalization has not been experimentally demonstrated.

Here we show that the pre-WGD ancestor of *SKI7* and *HBS1* was alternatively spliced. We also show that in most extant fungi, including ascomycetes and basidiomycetes, the *SKI7*/*HBS1* gene is still alternatively spliced, thereby describing the by far most conserved fungal alternative splicing event. The *L. kluyveri* alternative splicing isoforms are functionally distinct, such that one spliced mRNA encodes a functional Hbs1, while an alternatively spliced mRNA encodes a functional Ski7. Sequence analysis indicates that a very similar subfunctionalization occurred in an ancestor of the *Schizosaccharomyces* genus. Finally, while the *S. cerevisiae PTC7* gene encodes two differently localized proteins through intron retention, in a related species this gene is replaced by a pair of duplicated genes that arose form WGD. Thus, evolution of a fungal ancestral alternatively spliced gene into two subfunctionalized genes occurred at least three times: twice for the *SKI7/HBS1* gene, and once for *PTC7*. This further suggests that alternative splicing and gene duplication are not independent mechanisms to diversify the proteome, but instead are interrelated.

## Results

### The *Lachancea kluyveri SKI7/HBS1* gene provides a rare example of producing two fungal proteins through alternative splicing

**Figure 1:**
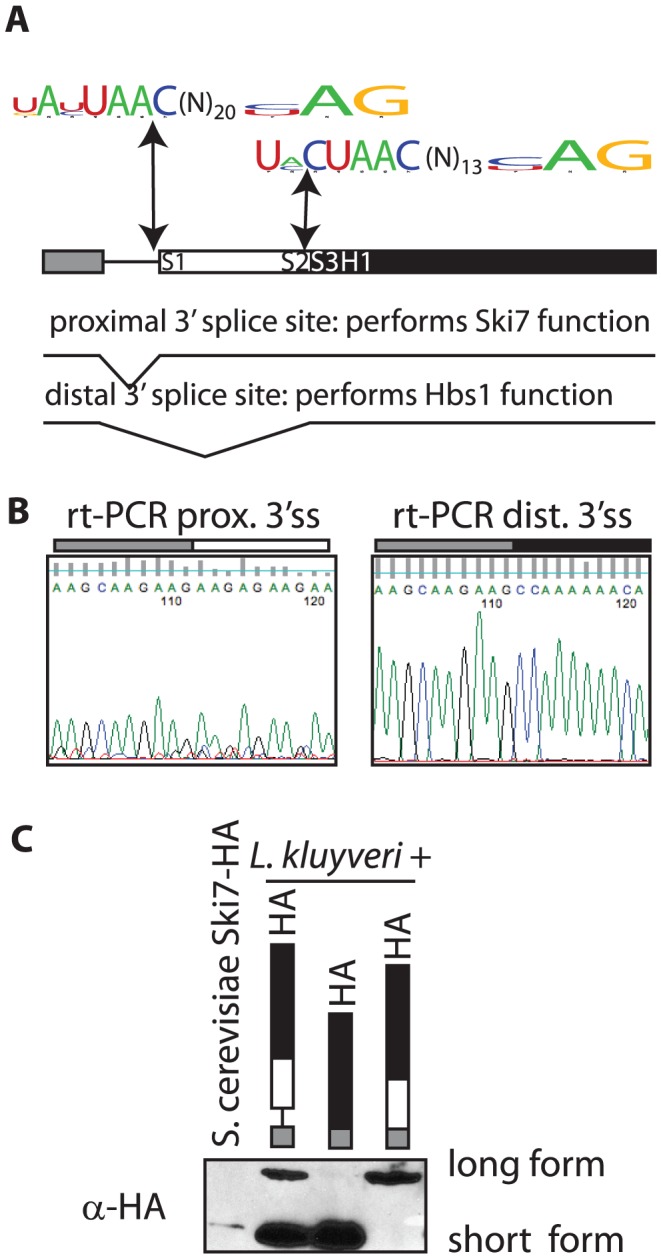
The *SKI7/HBS1* gene in pre-whole-genome duplication *Saccharomycetaceae* encodes two different proteins. A: Shown is the structure of the *L. kluyveri* gene drawn to scale. Exon 1 is indicated as a grey box, the exon that is only included in the long splice isoform is indicated as a white box, and the sequence downstream of the distal 3′ splice site is indicated as a black box. The sequence logos depict alternative 3′ splice sites shared with five pre-WGD *Saccharomycetacea*e. B: Sequencing rtPCR products confirmed that both *L. kluyveri SKI7/HBS1* 3′ splice sites are used. C: Western blotting shows that translation of the two splice isoforms of *L. kluyveri SKI7/HBS1* results in two distinct proteins. The first lane is a control *S. cerevisiae* strain with its *SKI7* locus HA-tagged. The second, third and fourth lane contain plasmids with either an alternatively spliced *L. kluyveri SKI7/HBS1* gene, or a modified gene with the intron removed as indicated.

Several genomes of *Saccharomycetaceae* have been sequenced, but incompletely annotated. Upon careful analysis of these sequences we noticed that the *SKI7/HBS1* genes in five pre-WGD *Saccharomycetaceae* each have a potential intron (Figure 1A). In contrast, *S. cerevisiae* and five other post-WGD species lack introns in both *SKI7* and *HBS1*. Furthermore, each pre-WGD gene has two potential 3′ splice sites, resulting in the potential to encode two different conserved proteins. In the case of *L. kluyveri* these proteins are predicted to be 70 and 96 KDa (Figure 1A). Although alternative splicing is rare in fungi, we speculated that the *SKI7/HBS1* gene may be alternatively spliced. We used rt-PCR to show that both predicted splice sites are indeed used. Use of the proximal 3′ splice site was confirmed using rt-PCR with a primer upstream of the 5′ splice site and a primer downstream of the proximal 3′ splice site and sequencing the resulting PCR product (Figure 1B left panel). Use of the distal 3′ splice site was similarly confirmed using a primer downstream of the distal 3′ splice site (Figure 1B right panel). Thus, the *L. kluyveri* gene is indeed alternatively spliced through the use of alternative 3′ splice sites.

To determine whether both spliced mRNAs were used to generate a protein, we generated a plasmid that introduced the HA epitope at C-terminus of the *L. kluyveri* ORF. A western blot shows that two proteins of the expected size are indeed made in *L. kluyveri* (Figure 1C; second lane). We further modified the HA-tagged plasmid by deleting the intron. In one construct we deleted sequences between the 5′ and distal 3′ splice sites, such that only the short 70 KDa splice isoform could be expressed. Figure 1C (third lane) shows that the encoded protein comigrates precisely with the smaller of the two species seen when the intron is included. Conversely, in another plasmid (fourth lane) we deleted sequences between the 5′ and proximal 3′ splice sites and as expected only the large 96 KDa isoform was made. Therefore, the rt-PCR and Western blot data show that the single *SKI7/HBS1* gene of *L. kluyveri* is used to generate two distinct proteins through use of alternative 3′ splice sites.

### The two proteins encoded by *L. kluyveri SKI7/HBS1* are functionally distinct

**Figure 2:**
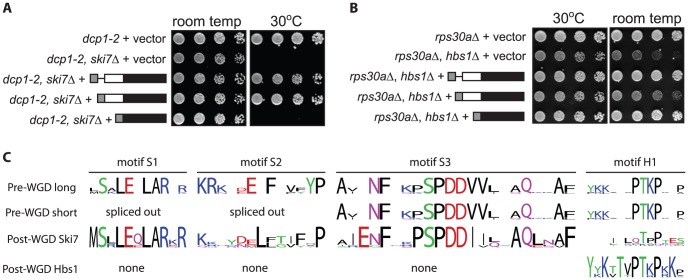
The two proteins encoded by *L. kluyveri* SKI7/HBS1 are functional. A: As shown before, *L. kluyveri SKI7/HBS1* can complement both the temperature sensitive phenotype of *dcp1-2 ski7Δ* (compare rows 2 and 3). In contrast, a plasmid encoding only the long splice isoform complements *ski7Δ* (Row 4), but the short isoform does not. B: Conversely, the cold sensitive phenotype of *rps30AΔ hbs1Δ* is complemented by the short isoform, but not the long isoform C: The pre-WGD *Saccharomycetaceae SKI7/HBS1* genes encode both sequences conserved in post-WGD *SKI7* but not in post-WGD *HBS1* (indicated as motifs S1, S2, and S3) and sequences conserved in post-WGD *HBS1* (N-terminal domain encoded by exon 1 and a short sequence motif indicated as motif H1) but not in post-WGD *SKI7* genes.

We have previously shown that the *L. kluyveri SKI7/HBS1* can carry out both the Ski7 and Hbs1 functions by showing that the *L. kluyveri* gene can complement both a *ski7Δ* and an *hbs1Δ* in *S. cerevisiae* [11]. To test whether both *L. kluyveri* proteins were generated in this context, we introduced the same HA-tagged constructs describe above into a wild-type *S. cerevisiae* strain. Western blot analysis indicates that when the *L. kluyveri SKI7/HBS1* gene is introduced into *S. cerevisiae*, both *L. kluyveri* proteins are made (data not shown). To determine whether one splice isoform carries out the Ski7 function and the other splice isoform carries out the Hbs1 function, plasmids expressing one or both proteins were introduced into both a *dcp1-2 ski7Δ* strain and an *rps30aΔ hbs1Δ* strain. A *ski7Δ* by itself does not result in a growth phenotype, but in combination with *dcp1-2*, results in a failure to grow at 30°C [26]. This *ski7Δ* phenotype can be complemented by the unmodified *L. kluyveri* gene (Figure 2A third row top panel) and by the long splice isoform (fourth row) but not by the short splice isoform (fifth row). Thus, only the long splice isoform can perform the Ski7 function. *hbs1Δ* by itself does not result in a growth phenotype but in combination with *rps30aΔ* results in slow growth at room temperature [27]. This *hbs1Δ* phenotype can be complemented by the unmodified *L. kluyveri* gene (Figure 2B third row bottom panel) and by the short splice isoform (5th row), but not by the long splice isoform (4th row). We conclude that alternative splicing generates two functionally distinct polypeptides, with the long splice isoform functioning similar to Ski7 and the short splice isoform similar to Hbs1.

Multiple sequence alignment (Figure S1) identified several sequence elements that correlated with Hbs1 function in short splice isoforms of pre-WGD *Saccharomycetaceae* SKI7/HBS1 genes and post-WGD HBS1 genes. The structure of *S. cerevisiae* Hbs1 has been solved, which shows a structured N-terminal domain and a C-terminal GTPase domain connected by a flexible linker [28], [29], [30]. The structured N-terminal domain is conserved in other post-WGD Hbs1 proteins but not in post-WGD Ski7. In the alternatively spliced pre-WGD homologs, this domain is encoded by exon 1, and thus present in both splice isoforms. The unstructured linker of Hbs1 is poorly conserved, with the exception of one sequence motif (Motif H1 in Figure 2C). A similar sequence motif is encoded in pre-WGD *SKI7/HBS1* genes, but has diverged very much in post-WGD SKI7 genes. The GTPase domain is also highly conserved in post-WGD Hbs1 proteins and pre-WGD Ski7/Hbs1 proteins (Motifs G1 to G5 in Figure S1), consistent with previous findings that Hbs1 GTPase activity is important for its function [31], [32], [33]. In contrast, Ski7 has not been shown to be an active GTPase and the domain has diverged rapidly post-WGD. Specifically, a catalytically important His residue in motif G3 is changed to Ser, Asn or Asp in post-WGD Ski7s.

The structure of Ski7 has not been experimentally determined, but the N-terminus is known to be important for interaction with the RNA exosome and three other Ski proteins [34]. Multiple sequence alignment indicated that although the Ski7 N-terminus is generally poorly conserved, it contains three conserved sequence motifs (Figure 2C and Figure S1; motif S1, S2, and S3). Alignment of pre-WGD Ski7/Hbs1 sequences shows that these motifs are also conserved in the pre-WGD species. Motif S1 and S2 are encoded between the two alternative 3′ splice sites and thus are only present in the longer splice form. These observations suggest that the short isoform of pre-WGD *SKI7/HBS1* genes may fail to carry out Ski7 function because they lack motifs S1 and S2.

### Usage of alternative 3′ splice sites in *SKI7/HBS1* is conserved in diverse ascomycetes

**Figure 3:**
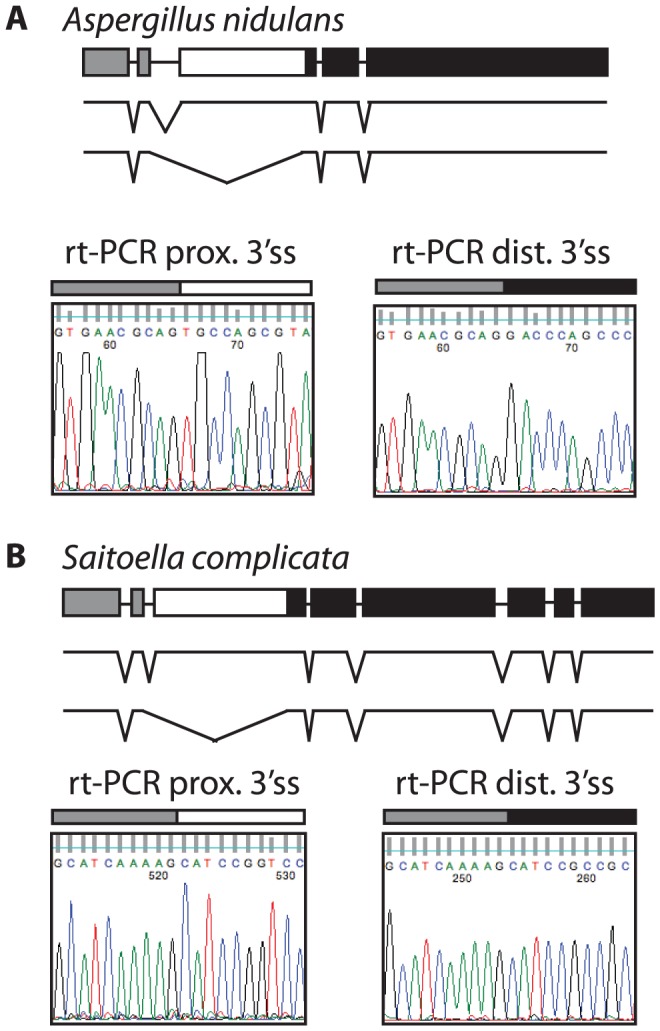
The use of alternative 3′ splice sites in *SKI7/HBS1* is conserved in the two other subphyla of *Ascomycota*. Shown are schematics of the *SKI7/HBS1* gene of *Aspergillus nidulans* (A; a representative of the *Pezizomycotina*) and of *Saitoella complicata* (B; a representative of the *Taphrinomycotina*), along with parts of the sequencing results of rt-PCR products confirming use of alternative 3′ splice sites. As in Figure 1, the alternative exon is indicated as a white box, while the upstream and downstream exons are indicated as grey and black boxes, respectively.

Most ascomycetes contain a single *SKI7/HBS1* gene. To determine whether alternative splicing of *SKI7/HBS1* was restricted to pre-WGD *Saccharomycetaceae* such as *L. kluyveri* or is a more ancient feature, we next looked at the more distantly related ascomycetes. The phylum *Ascomycota* can be divided in three subphyla, the *Saccharomycotina* (which includes *Saccharomyces* and *Lachancea*), the *Pezizomycotina* and the *Taphrinomycotina*. We therefore used the same rt-PCR and sequencing approach described above to analyze *SKI7*/*HBS1* splicing in *Aspergillus nidulans* and *Saitoella complicata*, which are members of the *Pezizomycotina* and *Taphrinomycotina*, respectively. Figure 3A shows that *Aspergillus nidulans* also uses alternative 3′ splice sites in *SKI7/HBS1* to generate two distinct mRNAs. The single *A. nidulans SKI7/HBS1* gene contains four introns. The second intron contains two predicted alternative 3′ splice sites, and rt-PCR and sequencing indicated that both are used. Similarly, the single *SKI7*/*HBS1* gene in *Saitoella complicata* contains seven introns, and the second intron contains two predicted 3′ splice sites. Figure 3B shows that both 3′ splice sites are used. Notably, the alternative spliced introns in *Lachancea*, *Aspergillus* and *Saitoella* are in the same position, just upstream of motif S3 characteristic of Ski7. Therefore, the capacity to use alternative 3′ splice sites in *SKI7/HBS1* is conserved throughout the phylum *Ascomycota*.

### Some fungi use different mechanisms to express distinct mRNAs from a single *SKI7*/*HBS1* gene

Although we were able to predict alternative 3′ splice sites in the single *SKI7/HBS1* gene of many other fungi, we noted four notable differences in the *Schizosaccharomyces* genus, a subset of the CTG clade (*Saccharomycetales* that use CUG as a serine codon instead of the canonical leucine), and the basidiomycetes *Cryptococcus neoformans* and *Ustilago maydis*. Species within the *Schizosaccharomyces* genus have duplicated *SKI7* and *HBS1* genes (see next section), while species in the CTG clade and the two basidiomycetes each contain a single *SKI7/HBS1* gene, but these genes lack obvious alternative 3′ splice sites.

**Figure 4:**
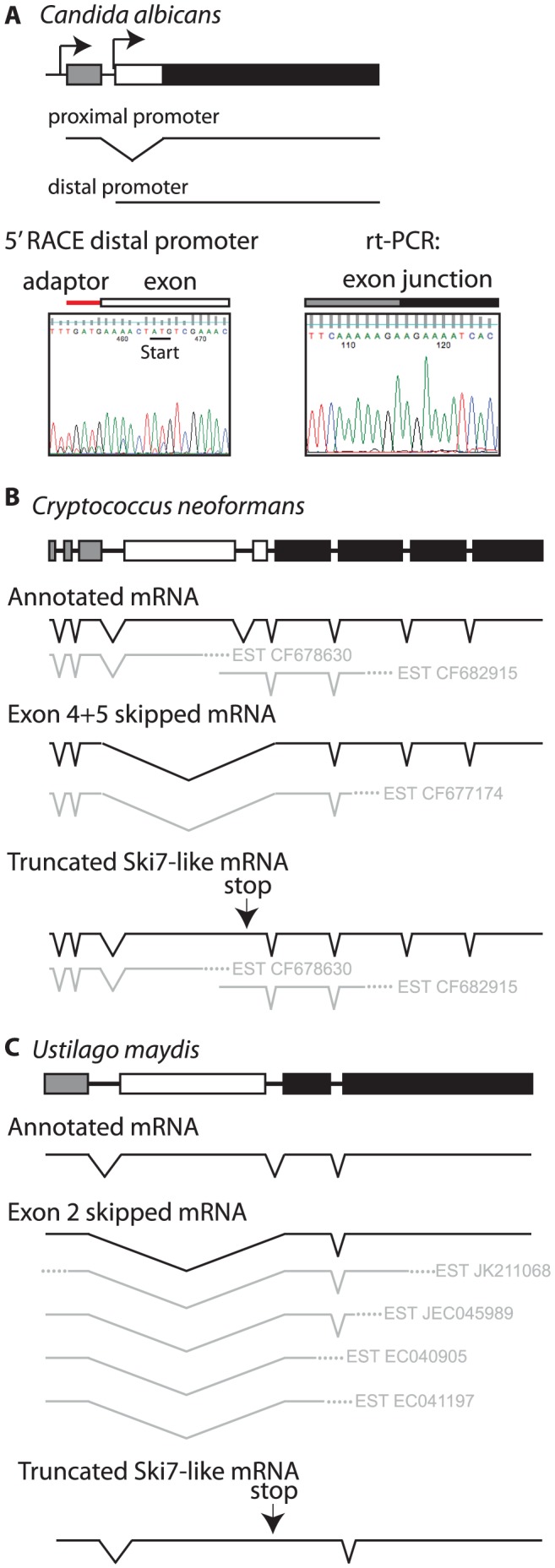
Some fungi use alternate methods to generate distinct *SKI7* and *HBS1* mRNAs from a single gene. Diagrammed are the gene structure of *Candida albicans* (A), *Cryptococcus neoformans* (B), and *Ustilago maydis* (C). Sequencing of rtPCR and 5′ RACE products confirms that *Candida albicans* generates distinct mRNAs through the use of alternative promoters, while EST sequences below the gene diagram show that basidiomycetes generates distinct mRNAs through exon skipping. As in Figure 1, the alternative exon is indicated as a white box, while the upstream and downstream exons are indicated as grey and black boxes, respectively.

The CTG clade can be divided into two smaller clades. One clade contains *Candida guilliermondii*, *Debaryomyces hansenii*, and *Candida lusitaniae*. In all three species there are two potential 3′ splice sites in locations similar to *L*. *kluyveri* (Figure S2). Thus, these three species appear to use the same mechanism as other ascomycetes to express two splice isoforms from a single SKI7/HBS1 gene. The other clade includes *C. albicans*, *C. dubliniensis*, *C. tropicalis*, and *C. parapsilosis*. These four *Candida* species also contain a predicted intron within their single *HBS1/SKI7* gene, however we only detected one potential 3′ splice site, which corresponds to the distal 3′ splice site of other ascomycetes. rt-PCR and sequencing confirmed that this 3′ splice site is used to generate an mRNA that is equivalent to the short splice isoform of *L. kluyveri* (Figure 4A). Although a proximal 3′ splice site is absent in these species, the capacity to encode Ski7-like sequence upstream of the distal 3′ splice site is conserved in these four species. In all four species motif S1 starts with a methionine. Since motif S1 is at the extreme N-terminus of the protein in post-WGD *SKI7* genes, we tested the hypothesis that the four *Candida* species generated a distinct mRNA that uses the AUG codon at the beginning of motif S1 as start codon. Figure 4A shows that 5′RACE indeed identified an mRNA with a 5′ end five nucleotides upstream of the conserved Ski7 motif S1. Thus, *C. albicans* uses alternative transcription start sites/first exons instead of alternative 3′ splice sites to generate two distinct mRNAs from the single SKI7/HBS1 gene.

The single *SKI7/HBS1* gene in basidiomycetes also appears to be alternatively spliced, although the details differ from the ascomycete situation. The *Cryptococcus neoformans* and *Ustilago maydis* genes have 9 and 4 annotated exons, respectively. Several EST sequences indicate that exons 4 and 5 in *C. neoformans* and exon 2 in *U. maydis* are skipped to generate an Hbs1-like protein (Figure 4B and 4C). Existing annotations suggest that a Ski7-like protein can be encoded by inclusion of these exons. However, EST, RNA sequencing and protein sequence similarity suggests an alternative where the annotated intron 4 of *C. neoformans* (and intron 2 of *U. maydis*) is not a true intron (See Text S1). This alternative mechanism encodes a truncated protein that resembles the N-terminus of Ski7, but is missing the GTPase domain (Figure 4B and 4C). This mechanism is strikingly similar to potential alternative splicing of the metazoan homolog (See Text S1 and Figure S5). Overall, while it is clear that the basidiomycetes use alternative splicing of their single *SKI7/HBS1* gene, it is not entirely clear which mechanism they use to generate a Ski7-like protein.

### Independent *SKI7/HBS1* duplication and loss of alternative splicing in the *Schizosaccharomyces* lineage

The fourth exception to conserved alternative 3′ splice sites in *SKI7/HBS1* occurs in the *Schizosaccharomyces* genus. The Hbs1 protein of *Schizosaccharomyces pombe* has been previously studied and appears to function similarly to *S. cerevisiae* Hbs1 [29]. In addition we found an uncharacterized paralog in *S. pombe* (Systematic name SPAP8A3.05) that encodes amino acid sequence motifs characteristic of Ski7p (labeled S1, S1′ and S3 in Figure S3). We therefore refer to this *S. pombe* gene as *SKI7*. Thus, an ancestor to *S. pombe* must have independently duplicated its *SKI7/HBS1* gene. The other three *Schizosaccharomyces* species with sequenced genomes each contain one clear ortholog of *HBS1* and one clear ortholog of *SKI7* (Figure S3), which suggests this duplication occurred before the *Schizosaccharomyces* species diverged from each other. Of the sequenced fungal genomes, the most closely related species with a single *SKI7*/*HBS1* gene is *S. complicata*. As discussed above, *S. complicata* has an alternatively spliced *SKI7*/*HBS1* gene, and thus the duplication in the *Schizosaccharomyces* genus appears to have occurred after it diverged from *S. complicata*, but before the *Schizosaccharomyces* species diverged from each other.

### *PTC7* provides a second example of post-WGD duplicate retention and loss of splice-isoforms

**Figure 5:**
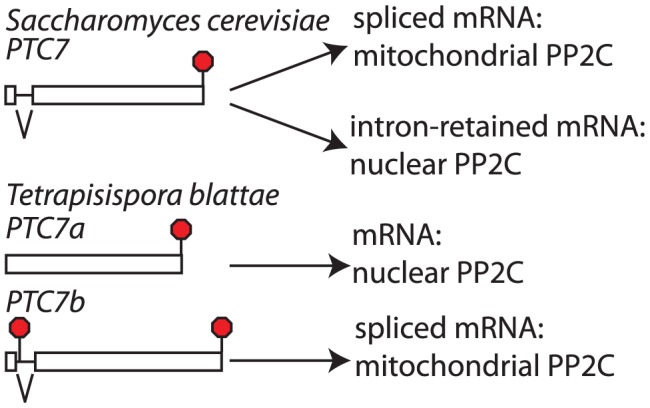
The *PTC7* gene is subfunctionalized through loss of alternative splicing in *Tetrapisispora blattae*. In *Saccharomyces cerevisiae* and most other *Saccharomycetaceae* the *PTC7* gene produces a nuclear envelope associated protein phosphatase through translation of the pre-mRNA and a mitochondrial protein phosphatase through translation of the spliced mRNA. In the post-WGD species *T. blattae*, both duplicated copies were maintained and one encodes a predicted nuclear envelope associated protein phosphatase, while the other encodes a predicted mitochondrial protein phosphatase.

Our above observations indicate that *S. cerevisiae SKI7* and *HBS1* evolved from a single alternatively spliced ancestral gene and that they correspond to the different splice isoforms of the pre-WGD ancestor. This conclusion suggests that a similar mechanism may apply to other alternatively spliced genes. The only *S. cerevisiae* gene known to use splicing to generate two different functional proteins is *PTC7* [20]. The *PTC7* gene contains an intron that can either be spliced out or retained. Both the spliced and unspliced mRNAs encode type 2C protein phosphatases (PP2C) [20]. It has previously been noted that an intron of 3n nucleotides without any in frame stop codons is conserved in the *PTC7* gene of twelve species within the *Saccharomycetaceae*, both pre- and post-WGD ([20]; Figure 5 and Figure S4). We searched for *PTC7* genes in additional yeast genomes and noticed that the only species within the *Saccharomycetaceae* that did not follow this pattern is *Tetrapisispora blattae*. The genome of this species contains two *PTC7* genes (which we will call *PTC7a* and *PTC7b*). The synteny pattern (http://wolfe.gen.tcd.ie/ygob/) indicates that after WGD the *T. blattae* lineage maintained both copies of *PTC7*, while one copy was lost in the *S. cerevisiae* lineage. We searched for potential introns in *PTC7a* and *PTC7b*, but failed to find one in the *PTC7a* gene, while *PTC7b* contains a 103 nucleotide intron. The spliced *PTC7b* mRNA is predicted to encode a functional protein. In contrast to other post-WGD species, translation of the *PTC7b* unspliced mRNA does not encode a functional PP2C: translation starting from the normal start codon would end after 20 amino acids at a stop codon within the intron, while the only other in frame AUG codon is only 8 amino acid upstream of the normal stop codon. Thus, unlike other *Saccharomycetaceae* that encode two proteins from one alternatively spliced *PTC7* gene, the *T. blattae PTC7a* and *PTC7b* genes each can only encode a single protein.

The two splice isoforms of *S. cerevisiae PTC7* are targeted to different compartments. The spliced *S. cerevisiae PTC7* mRNA encodes a protein that is localized to the mitochondria, while the intron-retained mRNA encodes a protein localized to the nuclear envelope. Targeting to the nuclear envelope has been attributed to a predicted trans-membrane helix (TM) that is encoded by the retained intron [20]. We used the TMHMM 2.0 server (http://www.cbs.dtu.dk/services/TMHMM/) to predict TMs in Ptc7 proteins of various *Saccharomycetaceae*. Each of the intron-retained mRNAs from post-WGD species and the *T. blattae PTC7a* gene encodes a single predicted TM near the N-terminus, suggesting that all of these proteins are targeted to the nuclear envelope. In contrast, none of the spliced mRNAs or *PTC7b* encode a predicted TM. We also used the PSORT II server (http://psort.hgc.jp/form2.html) to predict TMs and protein localization. The TM results agreed with the TMHMM server. In addition, all of the spliced isoforms and *PTC7b* were predicted to contain a mitochondrial targeting sequence that was absent from the unspliced isoforms. Thus, *T. blattae PTC7a* encodes a single PP2C that is predicted to be targeted to the nuclear envelope, like the protein encoded by intron-retained *PTC7* mRNA in *S. cerevisiae*, while *T. blattae PTC7b* gene appears to encode a single PP2C that is predicted to be targeted to the mitochondria, like the protein encoded by spliced *PTC7* mRNA in *S. cerevisiae*. While separate functions of the *S. cerevisiae PTC7* splice isoforms have not been defined, these results strongly suggests that the *PTC7a* and *PTC7b* genes are subfunctionalized, and thus that subfunctionalization by loss of splicing isoforms is not restricted to *SKI7/HBS1*.

## Discussion

### Conserved alternative splicing of fungal *SKI7/HBS1* genes

We describe alternative splicing in fungal *SKI7/HBS1* genes that is unusual in two respects. First, unlike in *Metazoa*, most fungal alternative splicing events do not produce two different proteins, but instead either have no known function or function to quantitatively regulate gene expression. Both of the mRNAs that are produced through *SKI7/HBS1* alternative splicing are predicted to encode functional proteins, western blot analysis indicates that both predicted proteins are produced, and complementation of *S. cerevisiae* mutants shows that the two proteins are functionally distinct. Second, most alternative splicing events that have been described in fungi are not widely conserved but instead have only been described in one species [MDH1, Ref. 22], genus [GND1, Ref. 21] or family [PTC7 Ref. 20]. Besides *SKI7/HBS1*, the most conserved fungal alternative splicing events were recently reported for *PGK1* in the Ascomycota and *GAPDH* in the Basidiomycota [23]. In contrast, alternative splicing of *SKI7/HBS1* most likely arose before the divergence of the *Ascomycota* from the *Basidiomycota*, and it might even have arisen before fungi and animals diverged (Text S1 and Figure S5). Thus, this alternative splicing event has been maintained for at least 500 million years. It has been suggested that the Ski7 function is peculiar to *S. cerevisiae* and close relatives [6], [35]. The finding of ancient alternative splicing indicates that Ski7 function is much older than appreciated and suggests that the ability to produce both Hbs1 and Ski7 is very important to fungi. Interestingly, one of the reasons why conserved alternative splicing in fungi has not been previously reported is that *S. cerevisiae* and *S. pombe* have been chosen somewhat arbitrarily as model fungi, and in both of these species the alternatively spliced *SKI7/HBS1* gene has been replaced with duplicate genes.

**Figure 6:**
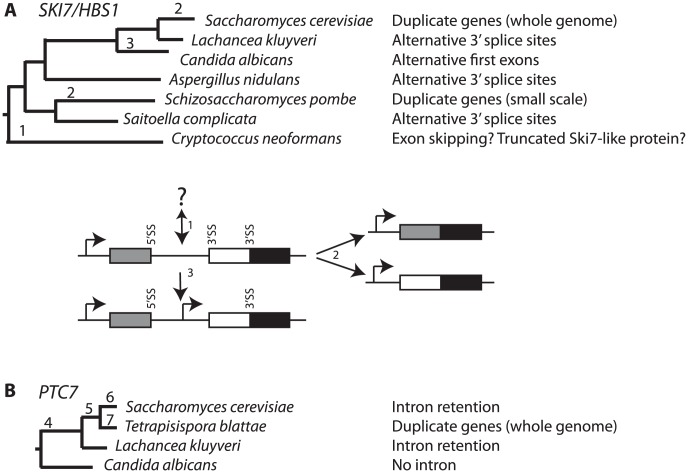
Changes in *SKI7*, *HBS1*, and *PTC7* gene structure during fungal diversification. *SKI7*, *HBS1* (A) and *PTC7* (B). See discussion for details. As in Figure 1, the alternative exon is indicated as a white box, while the upstream and downstream exons are indicated as grey and black boxes, respectively.

Although alternative splicing of *SKI7/HBS1* is conserved in diverse fungi, we have characterized changes in expression strategies for Ski7 and Hbs1, which are summarized in Figure 6A. The common ancestor of the ascomycetes and basidiomycetes appears to have had an alternatively spliced *SKI7/HBS1* gene. Although the exact nature of *SKI7/HBS1* alternative splicing event in basidiomycetes remains to be determined, it is clear that the mechanism by which a Ski7-like protein is expressed is different. Fully characterizing this event will require additional data from the basidiomycetes and/or additional early branching fungi. Independent duplications in the *Saccharomyces* and *Schizosaccharomyces* lineages allowed loss of alternative splicing (Figure 6 events 2). In the *Saccharomyces* lineage this duplication was part of a WGD, but in the *Schizosaccharomyces* lineage this duplication appears to be restricted to a single gene. The fourth evolutionary change occurred in the *Candida* clade in which a single *SKI7/HBS1* gene gained an alternative initiation codon for Ski7 (Figure 6 event 3). Interestingly, after duplication, the *S. cerevisiae*, and *S. pombe SKI7* genes also appear to have gained a new initiation codon (see below).

### Parallel precise post-duplication loss of the *HBS1* intron in budding and fission yeasts

A major mechanism for intron loss in *S. cerevisiae* involves a transposon-encoded reverse transcriptase that converts spliced mRNA into cDNA. This cDNA then recombines with the gene, resulting in precise deletion of the intron [36]. Multiple sequence alignment shows that the intron in post-WGD *Saccharomycetaceae* is precisely deleted (Figure S1), consistent with it being deleted by this mechanism. Similarly, the short isoform from *Saitoella complicata* aligns very well with Hbs1 sequences from four *Schizosaccharomyces* species, indicating that the alternatively spliced intron was precisely deleted (Figure S3). The *Saitoella SKI7*/*HBS1* gene contains seven introns. Recombination with a cDNA preferentially deletes introns near the 3′ end of the gene while introns near the 5′ end are more likely to be retained [36]. Consistent with intron loss by recombination with cDNA, *HBS1* genes from all four sequenced *Schizosaccharomyces* species contain two introns that correspond to the first two *S. complicata* introns. Thus, the alternatively spliced intron was precisely deleted in both the *Saccharomyces* and *Schizosaccharomyces* lineage, possibly by reverse transcription of the mRNA into cDNA and recombination.

### Parallel post-duplication loss of *SKI7* N-terminal exons and gain of a novel initiation codon in budding and fission yeasts

In contrast to *HBS1*, the intron in post-duplication *SKI7* genes was not precisely deleted. Multiple sequence alignment (Figure S1) of post-duplication *Saccharomycetaceae* showed that although the Ski7 N-terminus is generally poorly conserved, it contains three conserved sequence motifs (motif S1, S2, and S3). In post-WGD *SKI7* genes in both the *Saccharomycetaceae* and in *Schizosaccharomyces*, motif S1 is located at the extreme N-terminus of Ski7, starting with the Met translated from the start codon. This is most consistent with the model that after duplication *S. cerevisiae SKI7* lost exon 1 and gained a new initiation codon. Similarly, *Schizosaccharomyces* Ski7 appears to have lost exons 1 to 4, and gained a new initiation codon. This deletion of both the *SKI7* intron and the first exon(s) in *Saccharomyces* and *Schizosaccharomyces* is inconsistent with simple recombination with a cDNA.

It has previously been noted that a significant number of the genes that were duplicated and retained in *S. cerevisiae* after WGD are also duplicated in *S. pombe* [37], suggesting parallel subfunctionalization events in the two species. Our observations provide striking similarities of the evolution of the Hbs1 protein in *Saccharomycetaceae* and *Schizosaccharomyces* and of the Ski7 protein in these same clades and in *Candida albicans*. Thus, after independent duplication in these lineages, a similar sequence of changes occurred, intron deletion through recombination (*HBS1*) or generation of an alternative start site (*SKI7*).

### Generation of two Ptc7 proteins through splicing or gene duplication in *Saccharomycetaceae*

The only known example of a *S. cerevisiae* gene encoding functional proteins from both unspliced and spliced mRNA is *PTC7*. This capacity to encode two proteins is conserved in most *Saccharomycetaceae*, but not in other *Saccharomycetales* (including *Candida*, *Pichia* and *Yarrowia* species) [20]. The time of divergence of the *Saccharomycetaceae* has not been carefully defined, but estimates indicate that it preceded divergence of mice from humans 75 million year ago. Strikingly, only about 30% of alternative splicing events are conserved from mice to human. Therefore, although alternative splicing of *PTC7* is not nearly as well conserved as that of *SKI7/HBS1*, it still has been conserved for a longer period than many human alternative splicing events.

Since *PTC7* homologs outside the *Saccharomycetaceae* lack an intron in the same position, the alternatively spliced *PTC7* intron appears to have been gained by an ancestor of the *Saccharomycetaceae* (Figure 6B event 4). The PSORT server (http://psort.hgc.jp/form2.html) predicts that Ptc7 proteins outside the *Saccharomycetaceae* (*i.e*. from *Candida*, *Pichia*, and *Yarrowia* species) localize to the mitochondria. Thus, the gain of an intron and the alternative splicing of this intron provides the *Saccharomycetaceae* with a PP2C localized to the nuclear envelope. After WGD (Figure 6 event 5), one copy of *PTC7* was lost in the *Saccharomyces* lineage and the remaining copy maintained the capacity to encode two proteins (Figure 6 event 6). In contrast, in *T. blattae* both duplicated *PTC7* genes were maintained, but each lost the ability to encode two PP2C splice isoforms (Figure 6 event 7) and subfunctionalized into one gene for a mitochondrial PP2C and one gene for a PP2C in the nuclear envelope.

### Subfunctionalization of duplicated genes by loss of alternative splicing

Our combined bioinformatic and experimental analysis shows that alternative splicing and gene duplication may be interrelated events in a cycle that diversifies the proteome. In this cycle, gain of alternative splicing, duplication, and loss of alternative splicing and subfunctionalization result in functionally distinct paralogs. Although there have been some previous descriptions of subfunctionalization by loss of alternative splicing [e.g. ref 24], our findings extend these descriptions in three important ways. First, previous descriptions are generally limited to two closely related species and thus cover only part of the evolutionary history of the gene. The ever-increasing number of sequenced fungal genomes allowed us to analyze *SKI7/HBS1* and *PTC7* gene structure and expression in diverse fungi thereby identifying when alternative splicing arose and was lost. The whole cycle of gain of an alternative splicing event, duplication, and loss of alternative splicing can be observed in the *PTC7* gene of *Saccharomycetaceae*. In contrast, although we have not been able to identify when alternative splicing of *SKI7/HBS1* arose, we have described independent subfunctionalization events by loss of alternative splicing in the *Schizosaccharomyces* and *Saccharomyces* lineages.

Second, our observations suggest that subfunctionalization by loss of alternative splicing occurred very similarly in the *Saccharomyces* and *Schizosaccharomyces* lineages. Thus, unlike previously described isolated examples this phenomenon appears to have occurred multiple times.

Third, in most previously described cases of loss of alternative splicing in duplicated genes it was not clear whether the splicing isoforms have distinct functions, and thus it is not clear in those cases that subfunctionalization and loss of alternative splicing are causally linked. Similarly, lack of one *PTC7* splice isoform does not cause an easily identifiable phenotype under lab conditions, making it impossible to test whether the duplicate *T. blattae* genes can substitute for one but not the other splice isoform. In contrast, *SKI7* and *HBS1* have well-described functions, allowing us to demonstrate that the splice isoforms of *L. kluyveri SKI7/HBS1* are functionally distinct.

## Materials and Methods

### Strains

The *S. cerevisiae, C. albicans*, and wild-type *L. kluyveri* strains have been described [11], [38]. The *L. kluyveri ura3* mutant strain FM628 was a kind gift of Mark Johnston. The *S. complicata* type strain Y-17804 was obtained from the USDA ARS culture collection.

### Identification of a single SKI7/HBS1 gene of *S. complicata* and closing a gap in the draft genome sequence

A draft sequence of the *S. complicata* genome is available [39]. BLAST analysis using the *S. pombe* Hbs1 identified two non-overlapping contigs that encoded N- and C-terminal parts of a *S. complicata* homolog. Extensive BLAST analysis with other queries did not reveal additional homologs. We hypothesized that these contigs represented different parts of the same gene. We used PCR to close the gap between the contigs and sequenced the PCR product directly. The assembled sequence of the *S. complicata SKI7/HBS1* gene has been submitted to Genbank (Accession number JQ928880). Since exons proved difficult to predict due to their small size, we sequenced rt-PCR products to determine the gene structure depicted in Figure 3B, which was then used to align the encoded protein with *Schizosaccharomyces* homologs.

### RNA isolation

*L. kluyveri* and *S. complicata* were grown in YPD and RNA was extracted using our standard method for *S. cerevisiae*. *C. albicans* growth and RNA extraction was performed as described [40]. *Aspergillus* RNA isolated from strain R21 was a kind gift from Taylor Schoberle and Greg May (UT MD Anderson Cancer Center). When contamination with genomic DNA proved to be a problem, we treated the RNA with DNase (Promega).

### PCR, rt-PCR, and 5′RACE

rt-PCR was done using a commercial kit per manufacturer's instructions (Sigma-Aldrich). To close the gap between the two *S. complicata* contigs, we used the same kit but omitted the reverse transcriptase to amplify genomic DNA. 5′ RLM-RACE was done using a commercial kit per manufacturer's instructions (Invitrogen). All PCR, rt-PCR and RACE products were sequenced directly (Genewiz) and exactly confirmed the predicted splice sites.

### Plasmids

pAv231 has been described [11]. It contains the *L. kluyveri SKI7/HBS1* gene, including the promoter, intron and 3′UTR sequences. pAv844 and pAv847 have the long form and short form of the intron removed, respectively. They were generated by overlap PCR using the forward overlap oligonucleotides oAv963 (tgctcaaccaaagcaagaag aagagaagaaattatctaaactgg) for pAv844 and oAv965 (tgctcaaccaaagcaagaag ccaaaaaacaagctatctctaatttc) for pAv847 and their reverse complements oAv964 and oAv966. To generate the HA-tagged SKI7/HBS1 gene, a plasmid encoding the C-terminus and triple HA tag was chemically synthesized (by Genewiz). This fragment was used to replace the Bcl I to Bgl II restriction enzyme fragment of pAv231, generating pAv888, which contains the entire *L. kluyveri SKI7/HBS1* gene with a C-terminal triple HA tag. A Bam HI Xba I fragment of pAv888 was then used to replace the Bam HI Xba I fragment of pAv844 and pAv846 to generate pAv903 and pAv905. Thus, pAv903 encodes a C-terminally HA-tagged version of the long Ski7-like isoform of *L. kluyveri SKI7/HBS1*, while pAv905 encodes the tagged short Hbs1-like isoform.

### Western blot analysis

Plasmids carrying the HA-tagged *L. kluyveri SKI7/HBS1* gene, or empty vector controls, were transformed into *S. cerevisiae* or *L. kluyveri* strains using a standard method [41]. Transformants were selected on SC-URA, and then grown overnight in SC-URA. Total protein was isolated using the glass bead method and analyzed by western blotting using anti-HA antibodies (Roche). As a control for the western blotting we used a *S. cerevisiae* strain with the HA epitope integrated at the C-terminus of the endogenous SKI7 locus.

### Sequence analysis

Fungal *SKI7* and *HBS1* homologs were initially identified by BLAST in the sequenced genomes and the predicted proteomes from 14 *Saccharomycetaceae*, 10 species from the CTG clade, 4 *Pezizomycotina*, 5 *Taphrinomycotina* and 2 basidiomycetes. In none of the cases was the gene annotated as alternatively spliced, and in a number of cases introns were not annotated or incorrectly annotated and were corrected based on our rt-PCR analysis. Multiple sequence alignments of various subsets of protein sequences were generated with the help of the ClustalW (http://www.ch.embnet.org/software/ClustalW.html) BOXSHADE (http://www.ch.embnet.org/software/BOX_form.html), and WebLogo (weblogo.berkeley.edu/) servers. The species trees in Figure 6 and Figures S2 and S3 are adapted from [42], [43], and [44].

## Supporting Information

Figure S1 Alignment of pre- and post-WGD *Saccharomycetaceae* Hbs1 and Ski7 sequences. Conserved residues are red and blue. The amino acid encoded at the exon junction is in bold. Motifs conserved in Ski7 (S1 to S3), Hbs1 (H1) or GTPases (G1 to G5) are indicated below the alignment. The *S. cerevisiae* Hbs1 sequence is highlighted according to its structure N-terminal domain, yellow; unstructured region green; translation factor-like domain blue. Post-WGD sequences are from *Saccharomyces cerevisiae*, *S. paradoxus*, *S. kudriavzevii*, *S. bayanus*, *Naumovozyma castellii*, and *Candida glabrata*. Pre-WGD sequences are the long Ski7-like isoform from *Zygosaccharomyces rouxii*, *Lachancea waltii*, *L. thermotolerans*, *L. kluyveri* and *Kluyveromyces lactis* as well as the short Hbs1-like *L. kluyveri* isoform. Other pre-WGD species also are predicted to express similar short Hbs1-like isoform that are not included.
(PDF)

Figure S2 Top: Putative splice signals in SKI7/HBS1 genes from the Saccharomycetales. Bottom: Phylogenetic tree of the Saccharomycetales with key events indicated. WGD indicates Whole Genome Duplication.
(PDF)

Figure S3 A. Tree depicting the relationship between the *Taphrinomycotina* species based on trees from Liu et al 2009 and Rhind et al 2011 B. Alignments of pre- and post-duplication Ski7 and Hbs1 proteins of *Taphrinomycotina*. Conserved residues are red and blue. Amino acids encoded at exon junctions are in bold. Two conserved motifs similar to motifs S1 and S3 from Figure S1 are indicated below the alignment. Sequences are from *Schizosaccharomyces pombe* (pomb), *S. japonicus* (japo), *S. octosporus* (octo), *S. cryophilus* (cryo), and *Saitoella complicata* (Scom). For *Saitoella complicata* both the long Ski7-like and short Hbs1-like isoform are shown. For *S. pombe*, the previously determined structure of the C-terminal translation factor-like part is highlighted in blue. Sequence motifs similar to S1 and S3 of Figure S1 are highlighted below the alignment.
(PDF)

Figure S4 Multiple sequence alignment of post-WGD Ptc7s that can be translated from either the spliced mRNA or the unspliced pre-mRNA. Sequences are from *Saccharomyces cerevisiae*, *S. bayanus*, *Candida glabrata*, *Naumovozyma dairenensis*, *N. castellii*,, *Kazachstania africana*, *K. naganishii*, and *Tetrapisispora phaffii*, and *T. blattae*. Also included are the pre-WGD Ptc7 sequences of *Torulaspora delbrueckii* as a representative species that diverged just before WGD, and *C. albicans* and *Yarrowia lipolytica* as representative species that diverged before intron gain. TMHMM 2.0 was used to predict the transmembrane helices depicted in green. Amino acids encoded by exon-exon junctions are in bold.
(PDF)

Figure S5 Potential for alternative splicing in the human Hbs1L gene. The gene structure is indicated as in Figure 1, except that only the first 6 exons are shown. A previously annotated HBS1L protein is supported by 89 ESTs and is similar to Hbs1 along it's entire length. To generate this HBS1L the 5th exon is skipped. In addition 8 ESTs suggest that an alternative ORF is generated b including exon 5 as the last exon. This mRNA would encode a truncated protein that includes a conserved sequence motif that is similar to motif S3 of Ski7 described in Figure S1.
(PDF)

Text S1 Alternative splicing and subfunctionalization of *SKI7/HBS1* may also occur in plants and animals.
(DOCX)
