# Supplementary material for: Alternative Splicing and Subfunctionalization Generates Functional Diversity in Fungal Proteomes
Source: PLoS Genet. 2013 Mar 14;9(3):e1003376. doi: 10.1371/journal.pgen.1003376 (PMC3597508; doi:10.1371/journal.pgen.1003376)
Supplement: Figure S1 — Alignment of pre- and post-WGD Saccharomycetaceae Hbs1 and Ski7 sequences. Conserved residues are red and blue. The amino acid encoded at the exon junction is in bold. Motifs conserved in Ski7 (S1 to S3), Hbs1 (H1) or GTPases (G1 to G5) are indicated below the alignment. The S. cerevisiae Hbs1 sequence is highlighted according to its structure N-terminal domain, yellow; unstructured region green; translation factor-like domain blue. Post-WGD sequences are from Saccharomyces cerevisiae, S. paradoxus, S. kudriavzevii, S. bayanus, Naumovozyma castellii, and Candida glabrata. Pre-WGD sequences are the long Ski7-like isoform from Zygosaccharomyces rouxii, Lachancea waltii, L. thermotolerans, L. kluyveri and Kluyveromyces lactis as well as the short Hbs1-like L. kluyveri isoform. Other pre-WGD species also are predicted to express similar short Hbs1-like isoform that are not included. (PDF) [file pgen.1003376.s001.pdf]

```
Scer_Ski7     1 ------------------------------------------------------------------------------------------------------MSLLEQLARKRIEKSKGLLSAD
Spar_Ski7     1 ------------------------------------------------------------------------------------------------------MSLLEQLARKRVEKSKGLSNAD
Skud_Ski7     1 ------------------------------------------------------------------------------------------------------MSLLEQLARKRLEKSKGPSNTI
Sbay_Ski7     1 ------------------------------------------------------------------------------------------------------MSLLEQLARKRLEKSKDPSNIV
Cgla_Ski7     1 ------------------------------------------------------------------------------------------------------MSRLEELARQRRLKKAGSESSE
Ncas_Ski7     1 ------------------------------------------------------------------------------------------------------MSRLEQLARERALKNKNLGTSS
Zrou_long     1 --MVAYEDDDLMDYEEELPEFENEADLDEYLNDDEYELMNQLFPHVKKEMVDYQGWDNFALKKTIFETNFDPDESLKILKRSFKKKK---SEGSSSGGLSKLEQLARARAAQNNTPKTSA
Lwal_long     1 ---MSQFYDDDMDYEDEVPEFQDESEFDDYLNDEEYKLMSDMFPRAKKELADYQGWTNLAVKVTIFDHEFNFDEAMIELKRSLKKKK----TEEAEPKMSALERLARRRALDGKKATETK
Lthe_long     1 MPQYYDDDDMDTDLQDEVPEFRDEAEFDDYLNDEEYQLMSEMFPRAKKELADYVGWDNLSVKVAIFDHDFDFDNAMIELKRSYKKKK----SEETQVKMSALEQLARRKALSARKQNEAG
Lklu_long     1 --MAKYYDEDDMDYHSDVPEFQDESEFDDYLNDEEYGLMNDMFPRAKKEMADYQGWNNLAVKLAIFDQNFDFNQAMIELKRIYRKKQFAQPKQEEEKKLSKLEILARKRASAVDQKQTSS
Klac_long     1 -MSKFYDDDDAVDYDDDVPDFQDEDEFDDYLNDEEFELMNQLFPLAKEQLADYQGWDNLAVKVAIFDHNFELEPALVDLKRRFKKK-----EQKVEKKLSALELLARKRAEARNKASTMA
Scer_Hbs1     1 -----MAYSDYSDGADDMPDFHDEGEFDDYLNDDEYDLMNEVFPTLKAQLQDYQGWDNLSLKLALFDNNFDLESTLAELKKTLKKKKT--------------------------------
Spar_Hbs1     1 -----MSYSDYSDGADEMPDFHDEGEFDDYLNDDEYELMNEVFPTLKAQLQDYQGWDNLSLKLALFDNNFELESTLTELKKALKKKKT--------------------------------
Skud_Hbs1     1 -----MAYSDYSDGADDMPDFHDEGEFDDFLNDDEYELMNQVFPSLKEQLQDYQGWDNLSLKLALFDNGFDLESTLAELKITLKKKKT--------------------------------
Sbay_Hbs1     1 -----MAYSDYSDGADDMPDFHDEGEFDDYLNDEDYELMNEVFPPLKEELRDYHGCDNLSLKLALLDNDFDLESTVVELKRTLKKKKA--------------------------------
Cgla_Hbs1     1 --MADYDDDDYMS--DELPDFADEAEFDDYLNDEEYDLMNEVLPQAKKELSEYQGWDNLSVKLAIFENEFDLQAALTELKRKFKKKKP--------------------------------
Ncas_Hbs1     1 ---MPAYSDDDYEDDEGPYEFHDEAEFDDYLNDEEYDLLNQVLPHLKTQMKEYQGWNNLDLKLALFDNEFESDVAFNQLKKTFKKKKI--------------------------------
                                                                                                 exon junction      motif S1
Lklu_short    1 --MAKYYDEDDMDYHSDVPEFQDESEFDDYLNDEEYGLMNDMFPRAKKEMADYQGWNNLAVKLAIFDQNFDFNQAMIELKRIYRKKQFAQPKQEA-------------------------


Scer_Ski7    23 QSHSTSKSASLLERLHKNRETKDNNAETKRKDLKTLLAK------------DKVKRSDFTPNQHSVSLSLKLSALKKSNSDLEKQGKSVTLDSK----------------------ENEL
Spar_Ski7    23 QSQNTSKSASLLERLHKNRGTKDRNAEIRKKDLKSLLAK------------DKIKRSDCISDQHTISLSLKLSALKKSNSDLERQEKSVTPESK----------------------ENEF
Skud_Ski7    23 QSQNTSKGASLLERLHKSREAKGNYAASKKKDLKTLLAK------------DKIKKNDGTSGQHAFSLSLKLSPLKKSNSDLDKKGKLAASESA----------------------ESQS
Sbay_Ski7    23 QSQNTSKSASLLERLHRNREAKSTNGETKKKDLRSLLAK------------DRITKNDNTSNQHTFTLSSKLSALRKPYNDLDTKAKPVPSKSA----------------------ESQS
Cgla_Ski7    23 SSKNYEGTSKSASLLEKLRNRKKTASESPHNSEANT--------EIANHGSQAALNQTSGKVRSTPIKTSSSSTLGSKLRELRSRKKVEEAKQK-----------------------ESV
Ncas_Ski7    23 TSSEDKPKSRLFLLRSDKSLPS-----SSDSDPATNTSASSLLESLKSKRNNKSLSAILQEKRGQSKNHNDDSESKSPLGSLSSKLLSLKR-------------------------KPEE
Zrou_long   116 PNDEEDRSISLLSKLRNGTRGEQFPKRTLGERLRAS---------------QPSVSASTNTNGLVSKLAALKKPSTNEPSSLGSRLSALRKQAP-----------------------VRE
Lthe_long   117 SEKEADKSGKNLLSALQKKTPSPEQPKSTLAARLAALQKKS---------TMGLKGSKGESGNTNKKANTFSELLRNRWPDSS--PGENEHSKESKISLSSRLSALQKAKVSVPSKTASD
Lwal_long   114 TSVSTTPKAANLLSSLQKKRISESGRISQEGASSRSLLQRKG--------TSLSDMTHKPATSTGHDAFTAKSSLKDRLSSLANRVDSKSAASESKLSLSARLSVLKKARDSVQQEKPNS
Lklu_long   119 HTSNKSASLLSRLKGKTTPSRLGSFHDSTLSSHRSSEVESDAAKPSLALLMRSKQRRVHTSLEPTQSLPESRTETSPRSSSLAFLLAGRRRTVSPQNSLASKLMALKTERKVANGSKAEL
Klac_long   115 PATTKVSLLDKLKNGGGNGQNGNPVKTTVSVASR-----------------LQSLKRGPITTDNNSNGAATASFSQPKTHTLASKLASLRKSKP--------------EGQIIEKPEAEE
Scer_Hbs1    84 ------------------------------------------------------------------------------------------------------------------------
Spar_Hbs1    84 ------------------------------------------------------------------------------------------------------------------------
Skud_Hbs1    84 ------------------------------------------------------------------------------------------------------------------------
Sbay_Hbs1    84 ------------------------------------------------------------------------------------------------------------------------
Cgla_Hbs1    85 ------------------------------------------------------------------------------------------------------------------------
Ncas_Hbs1    86 ------------------------------------------------------------------------------------------------------------------------

Lklu_short   94 ------------------------------------------------------------------------------------------------------------------------


Scer_Ski7   109 PTKRKSPDDKLNLEESWKAIKEMNHYCFLKNDPCINQTDDFAFTNFIIKDKKNSLSTSIPLSSQNSSFLSLKKHNNELLGIFVP------CNLPKTTRKVAIENFNRPSPDDIIQSAQLN
Spar_Ski7   109 STKGKSFGVKLNVEESWDVINEINRYCLLKDDPCVNQADDFALTNFIMNGGKSASSTEILLSPSKSSSLSLKRHYDELLGIFVP------STLPKKSRNVAIENFNRPSPDDIILSAQLN
Skud_Ski7   109 PAKGRPSNVDTNLQDSWDVINEVNYYCSLKGNVRTNKSNDFAFTNFIISDKLK-------AASTSSSFLCLQKQYDELFTIFQP------STLPKKAHDKAIENFTKPSPDDIIQLAQLN
Sbay_Ski7   109 PVEKRSSNVNLDPEDPWDVLNEINYYFSLKDHERIKQTKCFAFTDFVSNNKSK-------ESDTLSSSLPIKKHYNELFTIFQP------STVPRTAHDNAIENFNKPSPDDIIQSAQLN
Cgla_Ski7   112 DVTPEIDSLPNGQSNKQSRDQLKQQYLVEMAEFKALQGRLNINIDSSKTIRRLSGHSKFLVNSRNKVHKLYKQKRDMLLTVFYP-------ARNKSARKKSIEGFHKPSPDDIVLAAQEQ
Ncas_Ski7   113 KTTDRDQRPSKTQEEDSKPLDAPNTWDLINRLQLQLHDSENHKSFNDYTEYQIMKLLSNGSLSRPRASQILKRNYDDLFTVYYP----------SNKKTKIRENFGEPSPDDIILNAQLL
Zrou_long   198 KPREKKEDLPPSQPPQRQP---LDPWEVIRAMRG---NANHPTPPQNNDSLVLATIVMKQFASDKPSKELQKRNYDEMFAPYYPPASFSNTNSVKAAKTKAVENFKKPSPDDVVLEAQSR
Lthe_long   226 VSKLQRKPENATKPVEKPLKMKDVSFESLISKFSLGASFDPPKCPNLNIGISKVVLTEEQ--PHKSRLSRLKRKREEVFTVFYP------DTNSQSAKKQAVSNFQQKSPDDVVLEAQKK
Lwal_long   226 DRNEIKKQE-GQVPKSPAPKPAIATYEQLVLIYNSQPSGTHLSQPNLQLGITRTILS-KQ--LYNFTTSRPKRKHDELFTVFYP------DTNNQAAKKQAVTNFQKPSPDDVVLDAQRK
Lklu_long   239 HTTPADEREEKPVIVHEEKTDPWSSFQKLRESQGQGNTSLHTHFQPPQLRITNIIVFSEPSAGIQTSEGKLKRKYDEIFGVFYP------NNNSRAAKKQAISNFNKPSPDDVVLTAQKK
Klac_long   204 TNVEVVIEENKIDHEKKDKDDPWVILNQLKDS-------AYVSNINEILAINKVPLYSGILEKLEFNSTTTKRKHEELFSVYYP------NTNNRVVKKQAIANFSKPSPDDVVINQQKA
Scer_Hbs1    84 ------------------------------------------------------------------------------------------------------------------------
Spar_Hbs1    84 ------------------------------------------------------------------------------------------------------------------------
Skud_Hbs1    84 ------------------------------------------------------------------------------------------------------------------------
Sbay_Hbs1    84 ------------------------------------------------------------------------------------------------------------------------
Cgla_Hbs1    85 ------------------------------------------------------------------------------------------------------------------------
Ncas_Hbs1    86 ------------------------------------------------------------------------------------------------------------------------
                                                                                              motif S2                     motif S3
Lklu_short   94 -------------------------------------------------------------------------------------------------KKQAISNFNKPSPDDVVLTAQKK
```

```
Scer_Ski7  223 AFNEKLENLNIKSVPKAEKKE-----------------------------------------PINLQTPPTESIDIHSFIATH-PLNLTCLFLGDTNAGKSTLLGHLLYDLNEISMSSMR
Spar_Ski7  223 AFNEKLENLNIKSPLKTEKKE-----------------------------------------SINLQTAPTESIDIHSFIATH-PLNLTCLFLGATNSGKSTLLGHLLYELNEISIPSMR
Skud_Ski7  216 AFNEKLENLNIKSAFNAKKNE-----------------------------------------LIELQTPPTESIDINSYIATH-PLNSTCLFFGVTTSGKTTLLGHLLYELNEISISSIR
Sbay_Ski7  216 AFNEKLVNLSIESKSKADKNE-----------------------------------------LIELQTPPIESIDVNSFIANH-PLNLTCLFLGDTSSGKSTLVGHILYELDEISMASIR
Cgla_Ski7  225 VFQAVTENVAQLSIKPIKIKER--------------------------------VSKRFTDNMTDQTKVQKQQLTVEDFINDRNATSFQVPMFGLPGSGKSTILGQLSFHLGLTTREDIR
Ncas_Ski7  223 AFNDVHEKVSKLALEADKKTKEP--------------------------------------IKLTKPTPPKNPIEVRKYVDEL-KPYLNFVMLGNESAGKSTIIGRLLEDSGLVRIDEIR
Zrou_long  312 ALDNVTENVSKLKVQEEKQAK-----------------------------------QQSQPKLATKPTKPTDPIDIQNYLSKG-KPHCSFVVIGHVDSGKSTLMGRVLYDLGVVDISHLR
Lthe_long  338 AFEDVEKTTKGVENIKISNPK------------AEDENEQGLDEEEDKPFVSEPTIRT--YRKALVPTKPKKPIDLEDHLQHK-KPHLSFVVLGHVDAGKSTLMGRLLYDVGAVDNKLIR
Lwal_long  336 AFEEVAKATKGVEKMTIETSE---------------SRLDDSEDDDYRPSIPEPVVRS--YKKAIVPTKPKKPIDLELHFAKK-KPHLSFVVLGHVDAGKSTLMGRLLFDVGAVDNKLIR
Lklu_long  353 AFTDVEKVANEVSKLSVNGGS------------DQDIDIESEESDDDRKPKAEPVTKT--YKKITTPTKPRNPVDIQSYVSSR-KPHLNFVVLGHVDAGKSTLMGRLLYDVGAVNYKLIR
Klac_long  310 AF-EVDKIKEKVADLSIQDKQ-------SSKTVEKEEDINDDDDDDEKLPFEEPVVKT--YKKVSVPTKPKNAINISSYLDNK-KPHLNFVVLGHVDAGKSTLMGRLLYDVGAVDTKLIR
Scer_Hbs1   84 PKKPIAAANGSANVTQKLANISISQQRPNDRLPDWLDEEESEGERNGEEANDEKTVQR--YYKTTVPTKPKKPHDISAFVKSA-LPHLSFVVLGHVDAGKSTLMGRLLYDLNIVNQSQLR
Spar_Hbs1   84 PKNPAAAANGTAKVTQKLADTSISEQRPNNGPPDWLNEEDSEDERHGEGANDEKTVQR--YYKTTVPTKPKKPRDISAFIESS-LPHLSFVVLGHVDAGKSTLMGRLLYDLNIVNQSQLR
Skud_Hbs1   84 PKKTVAAVNGTDSVVQKLSDVSISKPIASNGLPDWLDEDEIENERNGNGSDDEERAVQ-RYYKTTVPTRPKKSQDVSAYIKSA-LPHLSFVVLGHVDAGKSTLMGRLLYDLKIVNQSQLR
Sbay_Hbs1   84 PKKPTVTADDTSNVAQKLAGTSISKPTGHGLPEWLDEEEEEEGERKGSDAGDDGRLVQ-RYYKTTVPTKPKKPQDISAFIQSE-LPHLSFVVLGHVDAGKSTLMGRLLYDLKIVNQVQLR
Cgla_Hbs1   85 KKEVTPATTTTKKVTKDLG---------KLSLNQNKKQDDEWLDLDEPRKTEEEDDTKIVYKKVTVPTKPRKPIDIESYIQKS-KPHVSFVVLGHVDAGKSTLMGRVLQDVGAVDKTYIR
Ncas_Hbs1   86 AASTPPPATTTQPVKKLKESVAKLALSEKQKGKENDSSEWLEEDDDDHHIKEEDEPTVRTYKKVTTPTKPRKAVNIPEYLKTA-KPHLSFVVLGHVDAGKSTLMGRLLYDIGAVDTNHIR
                                                                                 motif H1                   motif G1
Lklu_short 117 AFTDVEKVANEVSKLSVNGGS------------DQDIDIESEESDDDRKPKAEPVTKT--YKKITTPTKPRNPVDIQSYVSSR-KPHLNFVVLGHVDAGKSTLMGRLLYDVGAVNYKLIR


ScerSki7   301 ELQKKSSNLDP----------SSSNSFKVILDNTKTERENGFSMFKKVIQVENDLLPPSSTLTLIDTPGSIKYFNKETLNSILTFDPEVYVLVIDCNYDSWEKSLDGPNNQIYEILKVIS
SparSki7   301 ELQKKSSNLDS----------IPSNSFKIILDNTKTERMNGFSMFKKIVQIKNNLLPPSSSLTLIDTPGNIQYFDKETVNSILTFDPEVYALVIDCNYDSWEKSLDGPNNQIYKILKVIS
SkudSki7   394 ELQKKVNSLSL----------PASNHFKIILDNTKLERENGFSMCRKIIQIENDLLPPSSSLTLIDTPGNIKYFEKETINSILTFNPDIFTLVIDCDYDSWEKSLDGLNNQIYEVLRIIS
SbaySki7   294 DLKKSVDNFDA----------TTSNHFKIIVDNTKTERESGFSMFKKTIQMENSLLPSSSSLTLIDTPGNSEFFNKETINSILTFNSDVFTLVIDCNYDSWEKSLDSPTNKIYEILRIIS
CglaSki7   313 VIKTDMERMHFRRIDKLDTRLLTTSPFCWVVDETTEERQFGHSNN----IKPLHIKYNDTDIIINEVPGSFDLCSTIETK---RYVGNSVIIVVSAELGDYEANFD-MKKRLIEKLIYCN
NcasSki7   304 SVKKELEKSK--------------LNAEMLYLSKLMEKKMSSTFS---------LDKNVSEFSAFDIPGDLKHLSSSIKA---IRQCTTAILTIDCNTDAFESAFN-MGSATIQHIYLCK
Zroulong   396 KLKRESEIVG-----------KSSFHLAWVMDQTPEERERGVTVS----VCTNDFETPSTRFTIVDAPGHRDFVPNAIAG---ISEADAAVLSIDCGTDAFESGFN-LDGQTKEHTLLAR
Lthelong   443 KLKKESEMIG-----------KSSFHLAWVMDQTSEERNRGVTVD----ICTSDFKTKDATFTIVDAPGHRDFVPNAIAG---VSQVDVAVLSIDCSTDAFESGFN-LDGQTKEHTLLAR
Lwallong   438 KLKRESELAG-----------KSSFHLAWVMDQTSEERARGVTVD----ICTSDFKTSKATFTIVDAPGHRDFVPNAIAG---VSQVDVAVLSIDCSTDAFESGFN-LDGQTKEHTLLAR
Lklulong   458 KLKKESEQAG-----------KGSFHLAWVMDQTSEERDRGVTVD----ICTSDFETDRATFTIIDAPGHRDFVPNAITG---ISQADAAVLTIDCCVDAFESGFS-LDGQTKEHTLLAR
Klaclong   420 KLQKESEMIG-----------KSSFHLAWVMDQTSEERNRGVTVD----ICTSDFATTKSSFTIVDAPGHRDFVPNAIVG---ISQADVAVLSVDCGTDAFESGFN-LDGQTKEHALLAR
Scer_Hbs1  201 KLQRESETMG-----------KSSFKFAWIMDQTNEERERGVTVS----ICTSHFSTHRANFTIVDAPGHRDFVPNAIMG---ISQADMAILCVDCSTNAFESGFD-LDGQTKEHMLLAS
Spar_Hbs1  201 KLQRESETIG-----------KSSFKFAWIMDQTNEERERGVTVS----ICTSHFSTQRANFTIVDAPGHRDFVPNAIMG---ISQADMAILCVDCSTNAFESGFD-LDGQTKEHMLLAS
Skud_Hbs1  202 KLQRESETMG-----------KSSFKFAWIMDQTSEERERGVTVS----ICTSHFSTERANFTIVDAPGHRDFVPNAIMG---ISQADMAILCVDCSINAFESGFD-LDGQTKEHMLLAS
Sbay_Hbs1  202 KLQKESETLG-----------KASFKFAWIMDQTNEERERGVTVS----ICTSHFSTEKANFTIVDAPGHRDFVPNAIMG---ISQADMAILCVDCNTNAFEKGFD-LDGQTKEHMLLAS
Cgla_Hbs1  195 KLKKESENIG-----------KGSFHLAWVMDQTKEERERGVTVS----ICTSDFDTPNANFTIVDAPGHRDFVPNAIAG---VSQADVAILSIDCGINAFESGFN-LDGQTKEHALLAK
Scas_Hbs1  205 KLKKESERIG-----------KGSFALAWVMDQTTEERERGVTVS----ICTSDFDTPKANFTIVDAPGHRDFVPNAIAG---VSQADVAVLSIDCGTDAFESGFN-LDGQTKEHTLLAK
                                                       motif G2                motif G3
Lklu_short 222 KLKKESEQAG-----------KGSFHLAWVMDQTSEERDRGVTVD----ICTSDFETDRATFTIIDAPGHRDFVPNAITG---ISQADAAVLTIDCCVDAFESGFS-LDGQTKEHTLLAR


ScerSki7   411 YLNKNSACKKHLIILLNKADLISWDKHRLEMIQSELNYVLKENFQWTDAEFQFIPCSGLLGSNLNKTENIT-KSKYKSEFDSINYVPEWYEGPTFFSQLYLLVEHNMNKIETTLEEPFVG
SparSki7   411 YLNEISACKKHLIILLNKADLISWDKQRLEMIQSELTYVLTENFQWKEAQFQFIPCSGLLGSNLNNVKKIT-KSKYKSEFDAINDVPEWYEGPTFFSQLYFLMELNMNKIETTLEEPFIG
SkudSki7   404 YLNENSAYKKQLIVLLNKADLISWDKQRLEMIQSELNYLLTETFQWKNTQFQFIPCSGILGSNLNNAENVVSKSKYKSEFDSINDVPEWYEGPTFLSHLYSLMEANMNKIETTLDEPFIG
SbaySki7   404 YLNMNSTYKKQLIVLLNKADLISWDKLRLEMIQSELQYMLTETFQWETTQLQFIPCSGLSGSNLNGANKVATKSKYKSEFDSINDVPEWYEGPTFLSHLYSLMEANMNKIETTLDEPFTG
CglaSki7   425 GVG-----VRRILTIINKMDLIDWDMDRYTVMKHELELIYQQ-VGIDILKCDFIGTSAITGEALTN------------DGDNRKRMFTKGSFSSLLNVLLSYKLQFKKEVLESSLVNSQE
NcasSki7   397 QAN-----IDNIIIMMNKMDTIDWDQGRYFQIKNELQSFLSR-LGFKKEQFTWIPSSGLYGQGIV--------------HSSYPKPQNWESCPTLSEKLISLTRSHS---DDNIADSPFF
Zroulong   497 SLG-----VGHIIVAMNKMDTVDWYQERFEQIRRELSSFFET-IGYRPEQISWIPCSGLTGANVV-------------KRTTHEMQ-NWYQGRSLVEELEYKAEQIEKSYQEDIINAPFL
Lthelong   544 SLG-----VRHIVVAMNKMDSVDWYEGRFEDIKFELRNFFED-IGIKDDQLSWVPCSGLTGEGVY-------------QKEYPMGQ-TWYKGPSLVGRLEEVSRELQKYSFEDISNTPFV
Lwallong   539 SLG-----VRHIIVAMNKMDSVDWYEGRFEDIKFELRNFFED-IGIKEEQLSWVPCSGLSGEGVY-------------ETEYPVGQ-TWYKGPSLVKKLELLAQSLQPKDLKEIEDSPFV
Lklulong   559 SLG-----ARHIVVAMNKMDHEGWYPTRFFDIKWELESFFKD-IGIKKEQVSWVTCSGLSGEGVY-------------NIKRPLGI-DWYNDPSLVDCLEDVAKKLNKDESSEAIEANFL
Klaclong   521 SLG-----IKHIVVAMNKMDSVSWYEGRFNDIRSELAVFFEE-IGFKGNDVSWVPCSGLSGEGIF-------------KTPYPPSQ-TWYQGPSLVGELENVALKLACVDESVTK-EPFL
Scer_Hbs1  302 SLG-----IHNLIIAMNKMDNVDWSQQRFEEIKSKLLPYLVD-IGFFEDNINWVPISGFSGEGVY-------------KIEYTDEVRQWYNGPNLMSTLENAAFKISKENEGINKDDPFL
Spar_Hbs1  302 SLG-----IHNLIIAMNKMDNVDWSQERFEEIKLKLLPYLVD-IGFCKDNISWVPISGFSGEGVY-------------KIGYTDEVRQWYNGPNLMSTLENAALKISKESEEITKEDPFL
Skud_Hbs1  304 SLG-----IHNLIIAMNKMDNVNWSQQRFEEIKSKLLPYLID-IGFCKDNICWVPISGFSGEGVH-------------KIGYTDDVEKWYNGPNLMSTLENAALKVSKENEITSKEDPFL
Sbay_Hbs1  304 SLG-----IQNLIIAMNKMDSVNWSQQRFEEIKSKLLPYLVD-IGFSGDNISWVPISGFSGEGVH-------------KIDYPNEVKQWYNGPTLMATLENCASKISKESVKTTEEDPFL
Cgla_Hbs1  296 SMD-----IKRVIVAMNKMDTVQWSHERYEDIKQKLVKFLYD-IGFTDNQLLWVPCSGFSGEGVY-------------KIPYPEDA-AWYTGPTVIQTLENVASDVGMVTYQEVKDQSFI
Scas_Hbs1  306 SMD-----VNNIVVAMNKMDSVNWSQERFMDIKYKLSAFFEE-VGFHEDQIKWVPVSGFSGQGVF-------------KIAYPKEQ-DWYEGKCLVETLEDVAQAIGTEAKKAVEDCPFL
                              motif G4                              motif G5
Lklu_short 323 SLG-----ARHIVVAMNKMDHEGWYPTRFFDIKWELESFFKD-IGIKKEQVSWVTCSGLSGEGVY-------------NIKRPLGI-DWYNDPSLVDCLEDVAKKLNKDESSEAIEANFL
```

```
ScerSki7    530 TILQSSV-----LQPIAEINYVSLKVLINSGYIQSGQTIEIHTQYEDFHYYGIVSRMKNSKQILETNTKNNISVGLNPDILEVLVKIHNT-EDFTKKQFHIRKGDIIIHSRKTNTLSPNL
SparSki7    530 LILQSSV-----SQPTAETHHVSLKVLIKSGYIQSGQTIEIHTQHEEIHYYGIITRMKKSKLILETNIKNNLTVGVKSDILEILVKIHNT-EDLAKKEFHIHKDDLIIHSRKANTLLPNL
SkudSki7    524 IILQNPI-----LQPTAEHNCVSLKVLIKSGYIQSGQTIEIHTHYEEVCHYGIITKMTKPKLTSSPNTKDYLPIGVHSDILEVFVKIHST-EELTKKQAHIRKNDLVISSRKASISSPYL
SbaySki7    524 IVLHNPM-----HPPSAESNYISLKVFIKSGYIQSGQTIEIHTPYEESSYYGIITKMIKPKLTLRSDTKNRSSVGVHSDILEIHVKIHHT-NEFTKKQIHIHKNDLIISPRKANTLSPNL
CglaSki7    527 QTLLDSEKLIAIQTGNKHEQQLSVPFYIERGLILKGQKLKINRTGLE----VVVKSIKESLRKKKS-----VNVAVVGQSVDLLFEPSEAITSSNINNILTSCNSPKLIETSE------F
NcasSki7    494 FSLTKKPRPAKLDVEDKRGDVYTLMGEVLSGSIQIGESMTIYPSKQS----VTVEKISKVNTEGNTKRILQKSIAIEMDQVILTVSNLYNDKDIRISDVAASIGHELLSSTSFQ-----T
Zroulong    597 FSITEVI-----SVNK--NEEVVVSGKVESGSIQPGETLNIFPSEQS----VVVNRITIDNNERK------VPVATKGDFAVLRLRNAFA-EIIEAGDLAASVGVE-IPVRNT------L
Lthelong    644 FSILEVS-----PGSK--ANEAVVSGRVESGHIQGGETITVFPSEQS----VLVDQILTGNEQTP------APVAIKGDFVSLKLRNAFY-DDIQGGDLAAVVGYD-IPSAQE------F
Lwallong    639 FSVMEVS-----PGNK--ANEAMIFGRVESGHIQSGETITIYPSEQS----VLVDQISSGNDHAV------TPVAVKGDFVSLKLRNAFY-EDIQSGDIAAIVGYD-IPSAQE------F
Lklulong    659 FSILDVS-----PTSK--NNEVIVSGKVEAGSIQPGETITIYPSEQS----VLVDSILSGNDRAS------VKIGVAGDFVMLKLREAYY-EDIQSGDLATTVGND-IPTAQE------F
Klaclong    620 FNILDVT-----PTSK--NTSAIISGKVESGTIQPGETITIYPSEQS----CVVDSILCGNDSQK------VDIALHGDFVQLKLHNAFP-EDIQGGDLASIVGFD-IPSSQE------F
Scer_Hbs1   403 FSVLEII-----PSKKTSNDLALVSGKLESGSIQPGESLTIYPSEQS----CIVDKIQVGSQQGQSTNHEETDVAIKGDFVTLKLRKAYP-EDIQNGDLAASVDYSSIHSAQC------F
Spar_Hbs1   403 FSVLEII-----PLKKTSNELALVSGKLESGSIQPGESLTIYPSKQS----CIVDRIQVGSQQGQSTNHEETDVAIKGDFVTLKLRKAYP-EDIQNGDLAASVDYPSVHSAQC------F
Skud_Hbs1   404 FSVLEII-----PLKKTNSELALISGKLESGSIQPGESLTIYPSEQS----CIVDKIQVGSQQGQSTNHEETGVAIKGDFVTLKLRKAYP-EDIQNGDLAASVDYPSVHSAQC------F
Sbay_Hbs1   404 FSILEII-----PLKKTNNELALVSGKLESGSIQPGESLTIYPSEQS----CIVDKIQVGSQQGQSTNHEETDVAIKGDFVTLRLRKAYP-EDIQDGDLAASVDYSSVHSAQV------F
Cgla_Hbs1   396 FSILESS-----ASNK--NEEAIISGKVESGTIQPGETLTIYPSEQS----VTVDKIIMGKEQAP------VPIAVKGEFVTIKLRHAHP-EDIQGGDIAASVEFD-IAAYQK------F
Scas_Hbs1   406 FSILEVI-----PSKK--NEEAIISGKLGSGSIQPGETITVYPSEQS----CVVDKILKGKNQDQ------VGIAIKGDFVTLKLRHAHA-EDIQGGDIAASVDYT-ISASQN------F

Lklu_short 423 FSILDVS-----PTSK--NNEVIVSGKVEAGSIQPGETITIYPSEQS----VLVDSILSGNDRAS------VKIGVAGDFVMLKLREAYY-EDIQSGDLATTVGND-IPTAQE------F


ScerSki7    644 PNTLKLLALRLIKLSIQTHALSDPVDLGSELLLYHNLTHNAVKLVKILGTNDISINPNQSLIVEVEIIEPDFALNVIDSKYITNN-IVLTSIDHKVIAVGRIACQ---
SparSki7    644 PNTLKLLSLRLIKLSFQTRALNGRLHLGSELLLYHNLTYSAVKLVKIHGTNDTSINPNQSLIVEVEIMEPNFALKVIDSKYVTNN-IFLTSIDHKVIAAGRIICQ---
SkudSki7    638 SNALKLSILPSMKLSIQTHLLNDPVNLGSELILYHDLMCKTVKLVKILGTNATSIISNQSIIVEVEITEPNFALNVINSEYITNY-IVLTTTDHKVVAVGDIACR---
SbaySki7    638 PNSLKLLNLRSMTLSIQTCLFSNPIVVDSELVLYHDLIYTTIKLMKIVGTNASSINPNQSLIIEVEIMAPDFALNVISSKYVTND-VVLTSTDHKIVAVGKIACQ---
CglaSki7    631 NMKMWSLKNCSKQLDFNNILIFCVGRFLNICISNVEQTENNEKLNNFQYIFCRACLKSSENMLAFEPGYESAKSFVLFFENRAIGFGELLLNDRSF-----------
NcasSki7    605 DMFIFETNKKPSIGIGSRGSLYRDGAVIPVKIKNLTSTQESAETSDQSQSKMPSNCNVN------IECETERPVVLLDHKNGKKCGNLVLYHEETIVATGTFI-----
Zroulong    692 KVQALTFQMSRPLLPGTPFMFFKGVNEQPARVSKLNSIIDKQDPSKIIKKKVKHLGSNQAAIFEIELVEKERWIPFLTSTQNRRMSRIVMRKEGRTIAAGTIIE----
Lthelong    739 SAQILTFNLDRPLLPGTSFILFRGSCEQPARVKKLVSVVDKSDPTKILKKKVRHLGSKQAAIIEIELVEKKRRVPMLTFGENKHLGRIVLRKEGKTIAAGVVKALEF-
Lwallong    734 TAQILTFKLDRPLLPGTSFMFFRGSCEQPARVKRLVSTVDKSNSEKILKKKVKHLGSNQAAIVEIELTEKKRRVPMLPFDENKHLARIVLRKEGRTIAAGIIKSLDL-
Lklulong    754 TAQLLTFKLDRPLLPGTSFMLFRGGCEQPARIKKLVSIVCKKDPKKILKKKVKHLGSDQAAIVEIELIEKKRRIPILTIEKSKHLGRIVLRKEGRTVAAGVVESLDF-
Klaclong    715 TSRLLTFRLDRPLLPGTSLMLFRGATEQPCRIKKLCCTVDKSNPSKVLKKKVKHLGSQQAAIVEIELVEKKRRIPMLTFEQNKKLGRVVLRKEGRTIGAALIKSLDY-
Scer_Hbs1   507 VLELTTFDMNRPLLPGTPFILFIGVKEQPARIKRLISFIDKGN--TASKKKIRHLGSKQRAFVEIELIEVKRWIPLLTAHENDRLGRVVLRKDGRTIAAGKISEITQ-
Spar_Hbs1   507 VLELTTFEMNRPLLPGTPFILFIGVKEQPARIKKLISLIDKDG--NLSKKKVRHLGSKQQALVEIELIEVKRWIPLLTARENDRLGRVVLRKDGRTIAAGKIFEISQ-
Skud_Hbs1   508 VLELTTFEMNRPLLPGSPFILFIGIKEQPARIKKLISLIDKSS--NVLKKKVRHLGSKQRAFVEIELIEVKRWIPLLTASENDRLGRVVLRKDGRTIAAGKISEISQ-
Sbay_Hbs1   508 ILELTTFEMNRPLLPGTPFILFIGVKEQPAKIKRLISLIDKDN--NVIKKKVRHLGSKQRALVEIELIEVKRWIPLLTASENDRLSRVVLRKDGRTIAAGKISEISE-
Cgla_Hbs1   491 NLRMLTFKMDRPLLPGTPFMLFRGVCEQPARISKLISLVDKDDFETVIKKKVRHLSSHQAAIVEIELTEKRRWIPLLTFSQNEHIGRIVCRKDGRTIATGTIMP----
Scas_Hbs1   501 QLQLLTFKMDRPLLPGTSFMLFRGVCEQPARISKLVSTVDKHNPEKILKKKIRHLGSNQAAIVEIELTERKRWIPMLTFKENRHLGRVVLRKDGRTIAAGSVLKIMNK

Lklu_short 518 TAQLLTFKLDRPLLPGTSFMLFRGGCEQPARIKKLVSIVCKKDPKKILKKKVKHLGSDQAAIVEIELIEKKRRIPILTIEKSKHLGRIVLRKEGRTVAAGVVESLDF-
```
