# Supplementary material for: Alternative Splicing and Subfunctionalization Generates Functional Diversity in Fungal Proteomes
Source: PLoS Genet. 2013 Mar 14;9(3):e1003376. doi: 10.1371/journal.pgen.1003376 (PMC3597508; doi:10.1371/journal.pgen.1003376)

Supplemental figure S2.

Splice signals in SKI7/HBS1 genes from Saccharomycetales					
species	5'ss	proximal bp	proximal 3'ss	distal bp	distal 3'ss
pre-WGD *Saccharomycetaceae*					
Lachancea thermotolerans	GUAUGU	UAUUAAC	UAG	UACUAAC	CAG
Lachancea waltii	GUAUGU	UAUUAAC	UAG	UACUAAC	CAG
Lachancea kluyveri	GUAUGU	UAUUAAC	CAG	UCCUAAC	UAG
Kluyveromyces lactis	GUAUGU	GACUAAC	CAG	UACUAAC	CAG
Zygosaccharomyces rouxii	GUAUGU	UAUUAAC	UAG	UACUAAC	UAG
CTG clade:					
CTG clade 1:					
Debaromyces hansenii	GUAUGU	UCCUAAC	CAG	UACUAAC	UAG
Candida lusitaniae	GUAUGU	GACUAAG	CAG	UACUAAC	UAG
Candida guilliermondii	GUAUGU	UUCCAAC	UAG	UACUAAC	AAG
CTG clade 2:					
Candida albicans	GUAUGU	none detected		UACUAAC	UAG
Candida dubliniensis	GUAUGU	none detected		UACUAAU	UAG
Candida tropicalis	GUACGU	none detected		UACUAAC	UAG
Candida parapsilosis	GUACGU	none detected		UACUAAC	UAG

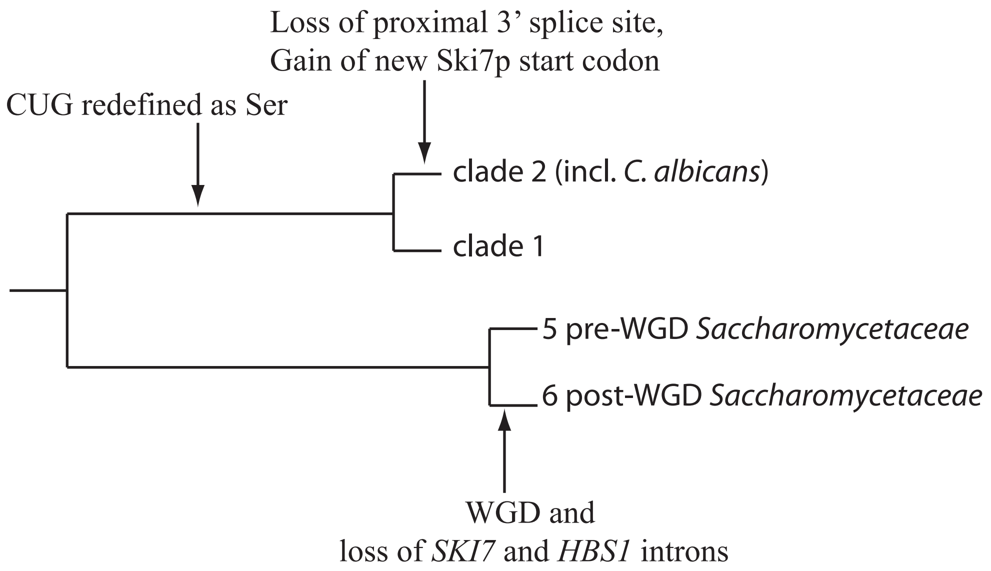

Supplement: Figure S2 — Top: Putative splice signals in SKI7/HBS1 genes from the Saccharomycetales. Bottom: Phylogenetic tree of the Saccharomycetales with key events indicated. WGD indicates Whole Genome Duplication. (PDF) [file pgen.1003376.s002.pdf]
