# Supplementary material for: Alternative Splicing and Subfunctionalization Generates Functional Diversity in Fungal Proteomes
Source: PLoS Genet. 2013 Mar 14;9(3):e1003376. doi: 10.1371/journal.pgen.1003376 (PMC3597508; doi:10.1371/journal.pgen.1003376)
Supplement: Figure S3 — A. Tree depicting the relationship between the Taphrinomycotina species based on trees from Liu et al 2009 and Rhind et al 2011 B. Alignments of pre- and post-duplication Ski7 and Hbs1 proteins of Taphrinomycotina. Conserved residues are red and blue. Amino acids encoded at exon junctions are in bold. Two conserved motifs similar to motifs S1 and S3 from Figure S1 are indicated below the alignment. Sequences are from Schizosaccharomyces pombe (pomb), S. japonicus (japo), S. octosporus (octo), S. cryophilus (cryo), and Saitoella complicata (Scom). For Saitoella complicata both the long Ski7-like and short Hbs1-like isoform are shown. For S. pombe, the previously determined structure of the C-terminal translation factor-like part is highlighted in blue. Sequence motifs similar to S1 and S3 of Figure S1 are highlighted below the alignment. (PDF) [file pgen.1003376.s003.pdf]

A.

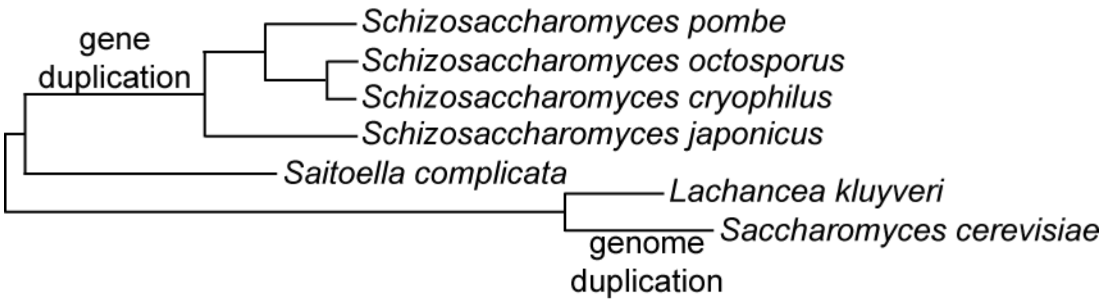

B.

```
pomb_Hbs1    1 MSRHRDVKNLDLDDYELDEEPG----EEELTEEQEEEFRSAVATVRETLLGVP-ISEKEIADTVWYYYFDVEKSVNYLLQKASSKAGAKEKQNTDSQKEKKQ------------------
japo_Hbs1    1 MSRHKDLKNLDLNDYEDEFEP-----EEELTEDQEEAYREAIEQVQTALEGTN-IPFQEIKDTVWYYYFDVNKSVNYLLSKA-IKAKEKDAKKTPVAQSQTK------------------
octo_Hbs1    1 MSRHRNVKNLELEEYDADEP------VEELTDEQEEQFREAIAAVQETVEGLP-VTNKEIADTVWYYYFDVEKSVNYLVKSCTARAEEKNGKDESHKKKPPK------------------
cryo_Hbs1    1 MSRHRNIKNLDLAEYDYEDEPA----QEELTDEQEEQFREAIAAVQETLDGLP-VTTKEIADTVWYYFFDVEKSINYLLKSCTTKAAAQKEKEESEKKKQNK------------------
scom_long    1 MSRHRDVRNLDLDEEMYDDDDYYDGDGHDMTYEEQEQMEAGVAAVHDALNGVPGITLKEIRETLYYYYFDLEKSIAWLLEQHSVKKPAAKPKAAPTASKASGPRLVIDEFDDEPPRKIAR
pomb_Ski7    1 ------------------------------------------------------------------------------------------------------------------------
japo_Ski7    1 ------------------------------------------------------------------------------------------------------------------------
octo_Ski7    1 ------------------------------------------------------------------------------------------------------------------------
cryo_Ski7    1 ------------------------------------------------------------------------------------------------------------------------

scom_short   1 MSRHRDVRNLDLDEEMYDDDDYYDGDGHDMTYEEQEQMEAGVAAVHDALNGVPGITLKEIRETLYYYYFDLEKSIAWLLEQHSVKKPAAKPKAAPTASKA

pomb_Hbs1   98 ------------------------------------------------------------------------------------------------------------------------
japo_Hbs1   96 ------------------------------------------------------------------------------------------------------------------------
octo_Hbs1   96 ------------------------------------------------------------------------------------------------------------------------
cryo_Hbs1   98 ------------------------------------------------------------------------------------------------------------------------
scom_long  121 ISASVIIARHGITWGMGSTASIEPVHPPRVPPGGWGLLGGSGKMSKLAALAKARKEAQ--------------------------------------------------------------
pomb_Ski7    1 -------------------------------------------MSRLSQLLNSKKAKQK--PPSEHPIGLSSILKQDSSSSSDSPNFFPSSSTNDHQERDTINDTNFVVPEKQKTSKLAL
japo_Ski7    1 -------------------------------------------MSKLAKLLSSKKAQKAENNTQQNEESKNVINS-------LTKSLETSSISGSSSNTQAQTQTRAPAVPSKLMSKLSQ
octo_Ski7    1 -------------------------------------------MSKLSQLLNAKRTNQQNSSLSESSGKLTSLLK---SPSSSSEQEHTASVDTVHTTSNAAAESSASTPQPNGLSKLAA
cryo_Ski7    1 -------------------------------------------MSKLSQLLNAKRANQQNTPAPESSGKLTSLLK---TPPPSVE-EHPAPLDFSQTTSNDTTEYSASSSQPSGLSKLAA
                                                            motif S1                                                    motif S1’
scom_short 101 ------------------------------------------------------------------------------------------------------------------------

pomb_Hbs1   98 ------------------------------------------------------------------------------------------------------------------------
japo_Hbs1   96 ------------------------------------------------------------------------------------------------------------------------
octo_Hbs1   96 ------------------------------------------------------------------------------------------------------------------------
cryo_Hbs1   98 ------------------------------------------------------------------------------------------------------------------------
scom_long  179 AASKAEKGAAPGLASVSLLSKLTAAKTAPKAAAAPDTPTEPPA-PQAPAEPAVSEPESSQPSSIPPPQSPSPPPVVADDVDMSEAPLLVSPTPSPSSPEHPTFEPALVEATPSVFATSLF
pomb_Ski7   76 LAAERKKLHSSFPSTQQQPPKTEKEKEKEPIQAKHKKNVENDFLLQRFRKVRIAEKKD----------SEQPSSHEIHLTDDDDKTTLQKQMVE--SDQLKKNPQEVKLAPPSSFAKCLT
japo_Ski7   71 LQKRKQQEQQEKHASLASETKNSQSNSKVNDTANLFSQFRKRRLNSQPTTPIIDSKSSLNTEPVPKATPLSPIPHN-TPSPPDRHNSQCSTSCY--DHSIQTPDASMYMSPPSSFAKCLC
octo_Ski7   75 LAAQKKKNENTEETANKRSLPSSSS---LQTDSNSKDSYDKEQLLQRFRKVRIAESKEPNEKPSLVHRQEQQTPTRDTKLNINEDDFASPSQTS--SAHLFSSSPTSLMASPSVFARCLT
cryo_Ski7   74 LAAQKK-NQNSEEASNKRPLPSSSSSS-LQPDTKSNDSFDKEQLLQRFRKVRIAESKTPSEELDHDTLSRYPAPPEEQVNNINYD--PSSSELP--QYDLSSSSPTSMMAAPSIFARCLT

scom_short 101 ------------------------------------------------------------------------------------------------------------------------
```

pomb_Hbs1 98 --NKSKEALADAKDPLDESSNGIKNLSLNKNDEPAFQTNGEVKMKNSSESDNQPEKKKIKKQNPTDLVSVPEIFEQSNPKPV
japo_Hbs1 96 ---ENSTKDVTAAVKKVTISQPSNALRKKQVIPQTTKTESEHAQIPQKKEPKLLDIPSLYEKEHEKPS
octo_Hbs1 96 ---QEQKQKQTVEAQPSTKNASASLNVSMKKLNLNGKEQTGEKSKDEEETQTPRKQESSLLIDVPKIYEESQPKPV
cryo_Hbs1 98 ---QEQNSTAGNRPSSKKTPVPLDASLEKLNLDGKEQTHETAKGLEETQKVRKQESSALIDVPKIYEESHPKPV
scom_long 298 GPEDYEVKYRSKRRREADSRFFYLPASAAPEVAKVFSGPSPDDVVIAAREEGPK**S**GKKAKTAKKDQAIAAPTNQLEVDMQAMNMGSSATGGAPAPAPPIKIARKKVNVVEEYAKVDVKES
pomb_Ski7 184 GAKKRVFEDQ-------------IEIHLSKSSLLGFNAPSPDDIVLMAQS---KSK**S**FQKHKRLDEQLLNSVKSMKKVSQQLKPQKNTNDSNNDHTLLSQDQ--LIELSKLV-----KPR
japo_Ski7 188 FDIHKPPKDN-------------IVIPYSRSALLGFHAPSPDDIVLAAQS---KSK**A**FQKTANK---------------------AAVGGAAQKPKEDVKLS--IEEITRHA-----KPV
octo_Ski7 190 GAKKKALQNE-------------IKVNLKRSSILGFNTPSPDDLVLMAQS---KSK**G**FQKHSKRDKALHDSLEQFKAANLQQASNSTTSPISTKDVLIDKEK--LLEISKEV-----KPT
cryo_Ski7 188 GAKKKAMQKE-------------IKVNLKRSSILGFNAPSPDDLVLMAQS---KSK**G**FQKYNRKDRALFNSLQQLKNASLQRTSTSIATPTVTKDVMVDKSK--LLELSKDI-----KPT

motif S3

scom_short 101 --------------------------SAAPEVAKVFSGPSPDDVVIAAREEGPK**S**GKKAKTAKKDQAIAAPTNQLEVDMQAMNMGSSATGGAPAPAPPIKIARKKVNVVEEYAKVDVKES

pomb_Hbs1 178 VHLVVTGHVDSGKSTMLGRIMFELGEINSRSMQKLHNEAANSGKGSFSYAWLLDTTEEERARGVTMDVASTTFESDKKIYEIGDAPGHRDFISGMIAGASSADFAVLVVDSSQNNFERGF
japo_Hbs1 161 IHLVVSGHVDSGKSTMIGRLLYEVGMVDERSMQKLKQSAANAGKGSFSYAWLLDSTDEERARGVTMDVADTTFESSKHIYQIGDAPGHKDFISGMIAGAYLSDYAILVVDASPNNFERGF
octo_Hbs1 169 VHLVVTGHVDSGKSTMLGRIMFELGDVNTRSMQKLHNEASNQGKGSFSYAWLLDSTDEERLRGVTMDVAATSFESHKQIYEVGDAPGHKDFISGMIAGAASAEFAILVVDSSQNNYERGF
cryo_Hbs1 169 VHLVVTGHVDSGKSTMLGRIMYQLGDVNSRSMQKLHNEAANQGKGSFSYAWLLDSTDEERLRGVTMDVAATSFESKKQIYEVGDAPGHKDFISGMIAGAASAEFAILVVDSSQNNYERGF
scom_long 418 ANFVII**G**HVDAGKSTMMGRLLYDIGAVDERTIQKFRKESEKMGKGSFALAWVMDSTDEERARGVTVDIATNQFETPKRKFTILDAPGHADFVPNMIAGAAQADFAVLVIDASTGGFESGF
pomb_Ski7 281 TKLLLLGPPKSGKKTLLSRLFFQIGSFDPKTMQKCTVLNAK--KESLSSVLKSTKTKWYDFETFSNSYSSTIIDFPLGIFTTNASS--RDNFLKHSSLFQVMNTAIFTIDCLNP-----L
japo_Ski7 264 IHIGIFGDVGAGKKSLLSRFLYQIGGLDTKHAQKCRLLNSR---QCTMESILSQSNDWYNFETFASSCSTTLLSYSEL------------DLNACSRRLFPLDMGVFVLRPHPTD-----
octo_Ski7 287 TRLSIFGPPKVGKKTLLARLLYQVGALDIKLMQRCALLNSR--KENFSSVLDRDASGLYQFETFSHNYLSSLFALPL------------QDLASFAPFLQTTDIVIVIIHAKYP-----L
cryo_Ski7 285 NRISIFGAPKVGKKTLLARLLYQVGALDIKLMQRCTLLNSR--KENLSSVLGRNESGLYQFETFSHNYLSSLFALPL------------HDLAALATFLQTTDIAVIVIHAKYP-----L

motif G1 **motif G2** **motif G3**

scom_short 195 ANFVII**G**HVDAGKSTMMGRLLYDIGAVDERTIQKFRKESEKMGKGSFALAWVMDSTDEERARGVTVDIATNQFETPKRKFTILDAPGHADFVPNMIAGAAQADFAVLVIDASTGGFESGF

pomb_Hbs1 298 LENGQTREHAYLLRALGISEIVVSVNKLDLMSWSEDRFQEIKNIVSDFLIKMVGFKTSNVHFVPISAISGTNLIQKDSSDLYKWYKGPTLLSALDQLVPP---EKPYRKPLRLSIDDVYR
japo_Hbs1 281 FSNGQTREHAYLLRALSVKGIAVCVNKLDTVDWSYERFIAIKENILDFLVSKVGFKETMVHIVPVSGLSGENLIKRDEPKLLSWYNGPTLMNILDDFVPP---TKPVKASLRITVNDTYR
octo_Hbs1 289 LANGQTREHAYLLRALGVSELAVAVNKLDLMSWSLDRYNEIKASVSDFLIRMVGFKEENVHFIPVSAVSGVNLIEKSSSPLYTWYNGPTLIEVLDNFNPP---LKAYRGPLRVSVHDTYR
cryo_Hbs1 289 LANGQTREHAYLLRALGVSEVAVAVNKLDLISWSSDRYFEIKSSISDFLVRMVGFKEENVHFIPVSAVSGVNLIEKQSSPLYNWYDGPTLIEVLDNFIPP---LKSYRGPLRVSVYDTYR
scom_long 538 NVRGQTKEHALLVRSLGVQNLIVAVNKLDSVDWNHERFEEIEMQVSQFLTN-AGFDPQNVQYIPCSGLTGENLVKRSAEPALTWFNGPTVLGAL**E**SIAPT---ARAIEKSLRISVQDVYK
pomb_Ski7 392 EGLDGISSILQLMNGLSISSYMFAITKMDEIEWDENKFINLVNSIQSFLKESCGIIEK-SKFIPISGLKGTNLTSISQEKLSQWYKSDTLLGKIDKEADTNHGTWNFLLNLPLSLTISHI
japo_Ski7 369 TELQHICEVVRVLDVLQIRLFVFIITGMDIVDWSESAFTETCQLLQQAIQKQCKQSIPATQFVPTSALNGENLTSLSQGKLRKWYRSGTVLSVLDELCESLAPQKKALNKEPLRASLESI
octo_Ski7 393 DEIESIVYLLRLCYGEKIRDVLFVVTQMDQVSWQEEQYQFAVSSITTCLKDTHGITVSPSSFIPVSGFKGDNVTTLSFGTLQSWYGSDTLLGKIDELSDNSTDEKIQLQHLPLSLSITSW
cryo_Ski7 391 DEIESIVCLLRLFYGVTIKNVLFVVTQMDQISWEEEQYQYVVSSVITCLKETYSITVPWSSFLPVSGFRGDNLTTLSYGTLQSWYDDETLLGKIDELSDKSIDEKLQLQHLPLSLTITSW

motif G4 **motif G5**

scom_short 315 NVRGQTKEHALLVRSLGVQNLIVAVNKLDSVDWNHERFEEIEMQVSQFLTN-AGFDPQNVQYIPCSGLTGENLVKRSAEPALTWFNGPTVLGAL**E**SIAPT---ARAIEKSLRISVQDVYK

pomb_Hbs1 416 S-------PRSVTVTGRVEAGNVQVNQVLYDVSSQEDAYVKNVIRNSDPSSTWAVAGDTVTLQLADIEVNQLRPGDILSNYENPVRRVRSFVAEIQTFDIHGPILSGSTLVLHLGRT--V
japo_Hbs1 398 S-------AKGVCVQGRVESGNVQNNQVLFNVSTNSDAYVKSIIRNGHP-HDWSVAGDNITMQLTDIDANEIRPGDILTTTSQPVKKTKSFIAEIQTFDLLRPILSGATLVLHRGRL--S
octo_Hbs1 406 S-------VRGATISGRVESGNIQNGQVLYNVSSQEDAYVKAVMRNSDPSVSWAVAGDNVTVQLTDIEPNQISLGDILSTYSDPVKRSKSFTADIQTFDILGPILSGSTLVLHRGRT--V
cryo_Hbs1 406 S-------VRGATVSGRVESGSIQNGQVLYNVSSQEDAYVKSVMRNSDPSVSWAVAGDSVTLQLTDIEPNQISLGDILSTYNDPVKRTKSFIAEIQTFDILGPILSGSTLVLHRGRT--V
scom_long 654 AGVT----GGSVTISGRVDAGNVQVGETVHAAPSGEPATVK**S**-MQVNDDIADWAVAGSNVVLNLNDIDPMHL**K**AGDILCDPLNPVPTVRAFRARIITFDLARPITNGATIVLHRGRINEA
pomb_Ski7 511 TPLP----ENQSHIYCSIHSGMLQDSQKLYVGTGRLETQIT**G**-LSSDENPKGFNVAGDMIQAKIPTLP--NL**C**PGILIADSIDAFTSSKTAYVNATWFHGSLEKGKSMHVIALFGCHAVL
japo_Ski7 484 QLEKTGESIGEVNATALIHSGILQQDMPFYLGAGKLACTVTTTHSITNSSHAFGVSGERVRLSFTLSSN-CPPVGSFISGDYAVTHSSRVLYFQGIALQPWMS-MDVHTMDLFVCGYHCK
octo_Ski7 508 SLLP----ENKIRAEFYVHSGILQAHQSIYTSVGKIETKVH**G**-IKLRQHERTWCLPGEQTEMHLTSLP--NL**L**NGTLCVDEENSYHLSRNAYITVFSIDSSLKAQNPVYVNAFFGAFRMS
cryo_Ski7 506 SLLP----ENKIAAQFDVHSGIVQAYQKIYTSVGKLEARVQ**S**-LKLHQHARTWCLPGEHTEMHLSSLP--NL**M**NGTLCIDVENSYHLSRIAYISAFTFDSSLKVHSPIYVNAFFGAFSMN

scom_short 431 AGVT----GGSVTISGRVDAGNVQVGETVHAAPSGEPATVK**S**-MQVNDDIADWAVAGSNVVLNLNDIDPMHL**K**AGDILCDPLNPVPTVRAFRARIITFDLARPITNGATIVLHRGRINEA

pomb_Hbs1 528 TSVSLKIVTVN-------NKRSRHIASRKRALVRISFLDGLFPLCLAEECPALGRFILRRS--GDTVAAGIVKELC-
japo_Hbs1 508 APVVIKLQSVN-------GKKVRHITSRKTAIVKFTFLDGSYPICTVDDCKTLGKIILRRE--GDTVAAGVIRKV--
octo_Hbs1 519 APVAVKIINVN-------GKKARHITSRKRASMQVNFLEGLYPVCTSEESPSLSRIILRRD--GNTIAAGIVKTIL-
cryo_Hbs1 519 ASVSVKIISVN-------GKKARHITSRKRASIQVNFLDGSYPVCPSEESSSLSRIILRRD--GNTIAAGIVKTIL-
scom_long 769 ARIQALVATIDRADGQIIKKKPRHLASGQSAVVEIAFLGNGIPMETFKDSKDLGRVILRTG--GDTIAAGIVDELF-
pomb_Ski7 624 TKLYCFTDSQEKA-----PNAIGNDLERNRTSLVKIELENAFPLVKESYINTLSRVLFVSEKWNSLIAFGTVLSLHD
japo_Ski7 602 ARLSIMSTERE----------STQQEDGPLELFKAEILDQPIVACTSSTLLSMSRIIAGNVDDHTVNVIGFITSML-
octo_Ski7 621 GKVFLYSEGKEND-----MALRGIEFHSKDLLLLRLELDQSVPLVEHNNVPSLSRLLLLSKEENALLASGVVLSVQK
cryo_Ski7 619 AKVFFYSEGKENT-----MALQGLQFQNKNLFLIRLELDQPIPLVEFNTVPSLSRVLLLSKEAKTLWASGIIISVQK

scom_short 546 ARIQALVATIDRADGQIIKKKPRHLASGQSAVVEIAFLGNGIPMETFKDSKDLGRVILRTG--GDTIAAGIVDELF-
