# Supplementary material for: Alternative Splicing and Subfunctionalization Generates Functional Diversity in Fungal Proteomes
Source: PLoS Genet. 2013 Mar 14;9(3):e1003376. doi: 10.1371/journal.pgen.1003376 (PMC3597508; doi:10.1371/journal.pgen.1003376)
Supplement: Figure S4 — Multiple sequence alignment of post-WGD Ptc7s that can be translated from either the spliced mRNA or the unspliced pre-mRNA. Sequences are from Saccharomyces cerevisiae, S. bayanus, Candida glabrata, Naumovozyma dairenensis, N. castellii,, Kazachstania africana, K. naganishii, and Tetrapisispora phaffii, and T. blattae. Also included are the pre-WGD Ptc7 sequences of Torulaspora delbrueckii as a representative species that diverged just before WGD, and C. albicans and Yarrowia lipolytica as representative species that diverged before intron gain. TMHMM 2.0 was used to predict the transmembrane helices depicted in green. Amino acids encoded by exon-exon junctions are in bold. (PDF) [file pgen.1003376.s004.pdf]

```
ScerPtc7-unspliced    1 --MFANVGFRTLRVSRGPLYGMFIVLFIGVLIAKFAGQMLI---DSETNFSHIIGSCSQIISFSKR-----TFYSSAKSGYQSNNSHGDAYSSGSQSGPFTYKTAVAFQPKDRDDL---IYQKLK
ScerPtc7-spliced      1 --MFANVGFRTLRVSRGPLYG----------------------------------SCSQIISFSKR-----TFYSSAKSGYQSNNSHGDAYSSGSQSGPFTYKTAVAFQPKDRDDL---IYQKLK
SbayPtc7-unspliced    1 --MFANVGFRTLRTSRGSIYGMFVILLLGVLIANFTGHLLI---DSESNISRIIGSCSQVINFSKR-----TFYSSAKDGYQSNGSNGDGYSANAQTGPFTYKTAVAFQPKDRDDQ---IYQKLK
SbayPtc7-spliced      1 --MFANVGFRTLRTSRGSIYG----------------------------------SCSQVINFSKR-----TFYSSAKDGYQSNGSNGDGYSANAQTGPFTYKTAVAFQPKDRDDQ---IYQKLK
CglaPtc7-unspliced    1 --MY-SAGVRVVNPWVLTKSMLIISLIMLIAGFLLTYTGLY----CPSYQIESGTLYNRVFGNCSR-----RLFFSGGKSWYWNGNNGSATTDHTGGNSFVYKTAVAYQPKDREDV---IYQKLK
CglaPtc7-spliced      1 --MY-SAGVRVVNPWVLTKK--------------------------------SGTLYNRVFGNCSR-----RLFFSGGKSWYWNGNNGSATTDHTGGNSFVYKTAVAYQPKDREDV---IYQKLK
NdaiPtc7-unspliced    1 --MFVSSGLRSLRGASVFSSSMFTRFAIALLLLSLLAHWLLI--QFPESPFHSTSTWKPLFPQLARRKSFFSTSSSSYYSHNNYSNSTNANGSEVSSSPLNYKTFVAYQPKDRDDQ---IYKNLK
NdaiPtc7-spliced      1 --MFVSSGLRSLRGASVFSSK--------------------------ESPFHSTSTWKPLFPQLARRKSFFSTSSSSYYSHNNYSNSTNANGSEVSSSPLNYKTFVAYQPKDRDDQ---IYKNLK
NcasPtc7-unspliced    1 --MFAATGLRTTVRQSPLLFYSMFTLLALALLIPILIPSL---------EYNFNRTCTQWYPLTKR--SFTSASGTNGNGHYNYSTNQSSSYS-TSASPFSYKTAVAYQPKDRDDQ---IYRNLK
NcasPtc7-spliced      1 --MFAATGLRTTVRQSPLLFYK----------------------------YNFNRTCTQWYPLTKR--SFTSASGTNGNGHYNYSTNQSSSYS-TSASPFSYKTAVAYQPKDRDDQ---IYRNLK
KafrPtc7-unspliced    1 --MLIIPEIRPFTLTSRTLYRMLLFIILTVFSGVVIDHLLI--QSDLGAAYINSPLHNRVFSLSKR-----FFTSNTAFNNSYTNGNGYSNTTNTTNPQFTYKAVVAYQPKDRNDQ---IYQKLK
KafrPtc7-spliced      1 --MLIIPEIRPFTLTSRTLYR---------------------------AAYINSPLHNRVFSLSKR-----FFTSNTAFNNSYTNGNGYSNTTNTTNPQFTYKAVVAYQPKDRNDQ---IYQKLK
KnagPtc7-unspliced    1 --MFAVGSARTLGATSRSYYGMFIFIVVTVFIAYMARLLIHPSFDDLSPVNNGFLLLKSQLGLISRRYFVSDSTHSNYQQYQGSGQQSSNANTGDISGPFSYKFAVAYQPKDRDDK---IYQKLK
KnagPtc7-spliced      1 --MFAVGSARTLGATSRSYYV-----------------------------NNGFLLLKSQLGLISRRYFVSDSTHSNYQQYQGSGQQSSNANTGDISGPFSYKFAVAYQPKDRDDK---IYQKLK
TphaPtc7-unspliced    1 --MFSVAGIRGSARICSNYGMYSQLLASILVLIFAVWILIPISSV--SLIAHVQYLKGNFYNLSKR--CFTSTGTFNDQSGNYSSYNYYTTSSQPQDTGLSYKLAVAYQPKDRDDP---IYKNLK
TphaPtc7-spliced      1 --MFSVAGIRGSARICSNYA-------------------------------HVQYLKGNFYNLSKR--CFTSTGTFNDQSGNYSSYNYYTTSSQPQDTGLSYKLAVAYQPKDRDDP---IYKNLK
TblaPtc7a             1 ---MNMSIMRANSRYYIFSISFVVLIVTFLFSTVL---------------------------------------KNYFNDWNLKDTIIKFTRYSNLEPNFHYVAEAAFQPKDRSSI---IYQKLA
TblaPtc7b             1 --MFCSSSLRPLRGSITSVYKISKISSHSATKLLIKRTFVN--------------GSWTTYNKSYSNYNSSNNSNSNDGGSSNSNYTHHHQLQDTPNNTFSYNTSVAYQPKDREDQ---IYKKLI
TdelPtc7-unspliced    1 --MFVSVGVRSARGPHVATNLWHSMFLFVVVSALVVVIAGYVLI--QGYEFDLEALSAQLYRGSRR--CFFS--------GKGDGYTGGDGSNGASSTQFSYKTAVAYQPKDRDDP---IYKRMK
TdelPtc7-spliced      1 --MFVSVGVRSARGPHVATNLWHK------------------------------ALSAQLYRGSRR--CFFS--------GKGDGYTGGDGSNGASSTQFSYKTAVAYQPKDRDDP---IYKRMK
CalbPtc7              1 MSMLLTILSKKNGVSLGSSFIKTS----------------------------ARSFASSSRSYWGGYGKGSARDYSTAASPSATASAASMNYDSALTSVSHYNIAVAFQPKDREESNLFKKKQPS
YlipPtc7              1 --MF--RPTRLPR------------------------------------------IQLLKPLFSRR--------------------------------FLSYTIAEAYSAKPRPPHQ--QSKPPA
consensus             1   mf   gvrtl       y                                   l   if  skr     t   s     q    ng   ss   s  ftYktavAyqpKdRdd    iyqklk

ScerPtc7-unspliced  113 DSIRSPTGEDNYFVTSNNVHD---------IFAGVADGVGGWAEHGYDSSAISRELCKKMDEISTALAENSSK---------ETLLTPKKIIGAAYAKIRDEKVVKVGGTTAIVAHFPSNGKLEV
ScerPtc7-spliced     82 DSIRSPTGEDNYFVTSNNVHD---------IFAGVADGVGGWAEHGYDSSAISRELCKKMDEISTALAENSSK---------ETLLTPKKIIGAAYAKIRDEKVVKVGGTTAIVAHFPSNGKLEV
SbayPtc7-unspliced  113 ESIKSPTGEDNYFTTSNNIHD---------IFAGVADGVGGWAEHGYDSSAISRELCKKMGELSTTLAEASSK---------ETLLTPKNIIDAAYARVKDEKIVKVGGTTAIMAHFPPNGKLQV
SbayPtc7-spliced     82 ESIKSPTGEDNYFTTSNNIHD---------IFAGVADGVGGWAEHGYDSSAISRELCKKMGELSTTLAEASSK---------ETLLTPKNIIDAAYARVKDEKIVKVGGTTAIMAHFPPNGKLQV
CglaPtc7-unspliced  111 ESLQSLTGEDNYFIQANAAND---------VYAGVADGVGGWAEHGYDSSAISRELCKALKEMAATLHK---------------PLTPKQLLDNAYAKIKIDKIVKVGGTTANVAHLSSDGRLDV
CglaPtc7-spliced     83 ESLQSLTGEDNYFIQANAAND---------VYAGVADGVGGWAEHGYDSSAISRELCKALKEMAATLHK---------------PLTPKQLLDNAYAKIKIDKIVKVGGTTANVAHLSSDGRLDV
NdaiPtc7-unspliced  119 DSLMSPTGEDNFFIASIDSND---------VYAGVADGVGGWAERNYDSSAISRELCRAMDQLATATLVSSKNQ------KYSDVISPKDLMDVAFEKIQNDKIVEVGGTTSIVAHFQKNGTLNV
NdaiPtc7-spliced     95 DSLMSPTGEDNFFIASIDSND---------VYAGVADGVGGWAERNYDSSAISRELCRAMDQLATATLVSSKNQ------KYSDVISPKDLMDVAFEKIQNDKIVEVGGTTSIVAHFQKNGTLNV
NcasPtc7-unspliced  109 DSLASPTGEDNYFITSLDNNDE--------IFAAVADGVGGWAERNYDSSAISRELCKAMGQLTSSSSIN-------------KVTTPKQVLEVSFQKIKDDKIVQVGGTTAIVAHFQKDGVLKV
NcasPtc7-spliced     90 DSLASPTGEDNYFITSLDNNDE--------IFAAVADGVGGWAERNYDSSAISRELCKAMGQLTSSSSIN-------------KVTTPKQVLEVSFQKIKDDKIVQVGGTTAIVAHFQKDGVLKV
KafrPtc7-unspliced  114 DSILSPTGEDNYFITSNSISD---------VYAAVADGVGGWAELGYDSSAISRELCNSMSKFTSTLSGRKDG------------ISPRDILDFAYNKIKEEGVVKVGSTTAIVAHFKDNGLLEV
KafrPtc7-spliced     89 DSILSPTGEDNYFITSNSISD---------VYAAVADGVGGWAELGYDSSAISRELCNSMSKFTSTLSGRKDG------------ISPRDILDFAYNKIKEEGVVKVGSTTAIVAHFKDNGLLEV
KnagPtc7-unspliced  121 DSLMSATGEDNFFVTSNSVSD---------LWTGVADGVGGWVEHGYDSSAISRELCKAMGQLASLPSPKGGK---------DQSLTPKDLIGSAYRKIKDEKTVEVGGTTAIAAHFENNGTLNI
KnagPtc7-spliced     92 DSLMSATGEDNFFVTSNSVSD---------LWTGVADGVGGWVEHGYDSSAISRELCKAMGQLASLPSPKGGK---------DQSLTPKDLIGSAYRKIKDEKTVEVGGTTAIAAHFENNGTLNI
TphaPtc7-unspliced  117 SSLDSPTGEDNYFVRKNANND---------VYVGVADGVGGWASYGYDSSAISRELCKAMSDYSTIK--NQKNS------LPFYEINPKTLIDISYNKIKDEKIVNVGGTTAIVGHFPPSGKLQL
TphaPtc7-spliced     88 SSLDSPTGEDNYFVRKNANND---------VYVGVADGVGGWASYGYDSSAISRELCKAMSDYSTIK--NQKNS------LPFYEINPKTLIDISYNKIKDEKIVNVGGTTAIVGHFPPSGKLQL
TblaPtc7a            81 NSVDSPTGEDSYFIASNSYND---------IYIGVADGVGGWAERGYDSSAISRELCSSMKALCR----------------AQTELTPKQLLSKGYNKIKSDGIVKVGSTTANVAHLTRNGILNV
TblaPtc7b           107 ASKKSPTGEDNLFINCSSLNDE--------VFAAVADGVGGWAEYGFDSSAISRELCENLNVFSNSFFQLQTTN--------AVTKAPKELLDLAYLKTKKDGIVEIGSTTALVAHLDPKGCLQI
TdelPtc7-unspliced  109 TQLQSATGEDNYFVTLNNPGD---------VYAGVADGVGGWAEHGYDSSAISRELCRAMNDFSSLS--NKKD---------SHAFPPKKLIEMGYNKIKNDGIVKVGGTTAIAAHFPSNGTMQV
TdelPtc7-spliced     81 TQLQSATGEDNYFVTLNNPGD---------VYAGVADGVGGWAEHGYDSSAISRELCRAMNDFSSLS--NKKD---------SHAFPPKKLIEMGYNKIKNDGIVKVGGTTAIAAHFPSNGTMQV
CalbPtc7             98 PSLQSPSGEDNLFVSNEKAG-C--------IAVGVADGVGGWSEAGYDSSAISRELCASLRRQFESG----------------TESNPKQLLSLAFKEVLSSPQVEIGGTTACLGVLTSDLKLHV
YlipPtc7             46 GENRPPTGEDAFFHVSLSKTDSPDSYTYSNTAFGVTDGVGGWAEMGVNSSDFSYYLCHESSNLAVEKAKEIEKEPAFAEKPLASLISPKQLLTNAYNKIVREKTVKAGGSTACIGVAGQDGQVAV
consensus           126 dsl sptGEDnyFvtsn   d         vyagVaDGVGGWaehgydSSaiSreLCkam  ist                    ltPk lid ay kikdekiVkvGgtTaivahf  ng lqv
```

```
ScerPtc7-unspliced  220 ANLGDSWCGVFRDSKLVFQTKFQTVGFNAPYQLSIIPEEMLKEAERRGSKYILNTPRDADEYSFQLKKKDIIILATDGVTDNIATDDIELFLKD---NAARTNDELQ-------------LLSQK
ScerPtc7-spliced    189 ANLGDSWCGVFRDSKLVFQTKFQTVGFNAPYQLSIIPEEMLKEAERRGSKYILNTPRDADEYSFQLKKKDIIILATDGVTDNIATDDIELFLKD---NAARTNDELQ-------------LLSQK
SbayPtc7-unspliced  220 ANLGDSWCGVFRDSKLVFQTEFQTVGFNAPYQLSIIPEKMLKEAERKGSKYILNTPADADEYTFQLEKNDIVMLATDGVTDNIAADDIELFLKD---NSARTKNELQ-------------LLSQE
SbayPtc7-spliced    189 ANLGDSWCGVFRDSKLVFQTEFQTVGFNAPYQLSIIPEKMLKEAERKGSKYILNTPADADEYTFQLEKNDIVMLATDGVTDNIAADDIELFLKD---NSARTKNELQ-------------LLSQE
CglaPtc7-unspliced  212 TNLGDSWCAVFRDSKLVFQTEPQTLGFNTPYQLAIIPDEIQQAAAKNGNRYIQNQPSDADEYNFQLSKGDIVILATDGVTDNIAIEDLELFLRD---NNDQLNENLQ-------------KTADE
CglaPtc7-spliced    184 TNLGDSWCAVFRDSKLVFQTEPQTLGFNTPYQLAIIPDEIQQAAAKNGNRYIQNQPSDADEYNFQLSKGDIVILATDGVTDNIAIEDLELFLRD---NNDQLNENLQ-------------KTADE
NdaiPtc7-unspliced  229 ANLGDSWCGVFRNYKLVFQTKFQTVGFNAPYQLSIIPKHLLEEARLKGTSYIRNTPADVDEYSFQLSQNDIVILATDGVTDNISTDDISLFLKDNS-EKLSTSKELN-------------AMTKD
NdaiPtc7-spliced    205 ANLGDSWCGVFRNYKLVFQTKFQTVGFNAPYQLSIIPKHLLEEARLKGTSYIRNTPADVDEYSFQLSQNDIVILATDGVTDNISTDDISLFLKDNS-EKLSTSKELN-------------AMTKD
NcasPtc7-unspliced  213 ANLGDSWCGVFRNETLVFQTKLQTVGFNAPYQLSIIPDSLLKEAALKGSSYIQNVPSDADEYTFQLQKNDIVMMATDGVTDNIITDDISLFLKD---ESAQIQKNLQ-------------NVTEK
NcasPtc7-spliced    194 ANLGDSWCGVFRNETLVFQTKLQTVGFNAPYQLSIIPDSLLKEAALKGSSYIQNVPSDADEYTFQLQKNDIVMMATDGVTDNIITDDISLFLKD---ESAQIQKNLQ-------------NVTEK
KafrPtc7-unspliced  218 ANLGDSWCGVFRDSKLVFETKFQTVGFNAPYQLSIIPDSISK-----GQKYIQNTPADADNYSFQLQKNDVILLATDGVTDNIGTEDMELFLKD---NEDQILQDLE-------------SVSKD
KafrPtc7-spliced    193 ANLGDSWCGVFRDSKLVFETKFQTVGFNAPYQLSIIPDSISK-----GQKYIQNTPADADNYSFQLQKNDVILLATDGVTDNIGTEDMELFLKD---NEDQILQDLE-------------SVSKD
KnagPtc7-unspliced  228 ANLGDSWCGVFRDHKMVFQTKFQTVGFNAPFQLAIIPEPMAKEAARTGRSYIQNTPEDADEYKFDLAKNDVVILATDGVTDNIDTGDIELFLRD---NEAQVETDFQ-------------NAAKE
KnagPtc7-spliced    199 ANLGDSWCGVFRDHKMVFQTKFQTVGFNAPFQLAIIPEPMAKEAARTGRSYIQNTPEDADEYKFDLAKNDVVILATDGVTDNIDTGDIELFLRD---NEAQVETDFQ-------------NAAKE
TphaPtc7-unspliced  225 ANLGDSWCGVFRDYKLVFKTNFQTVGFNAPYQLAIIPKELLSGKEN---SYIQNKPSDADEYTFQLEKDDIILLATDGVTDNIATGDMENFFRD---NEASTEEELQ-------------TITKK
TphaPtc7-spliced    196 ANLGDSWCGVFRDYKLVFKTNFQTVGFNAPYQLAIIPKELLSGKEN---SYIQNKPSDADEYTFQLEKDDIILLATDGVTDNIATGDMENFFRD---NEASTEEELQ-------------TITKK
TblaPtc7a           181 ANLGDSWCGVIRDSKIVFQTKFQTVAFNAPYQLSVIPDFILEEAKKLGSSYIMNIPLDADEYSFQLQKEDIVLLATDGLVDNIEPNDIALFISN----RFASKDNSK-------------SIVQS
TblaPtc7b           216 ANLGDSWCGVFRDNKLIFQTENQLLGFNTPFQLSIIPDSFLKARNQNKNSYIQNLPSDADEYSFQLKPNDIVILATDGVTDNIATGDIELYLKDNYDNKQLNNKELQ-------------DLTSK
TdelPtc7-unspliced  214 ANLGDSWCGVFRDSKLVFQTRFQTVGFNAPYQLAIIPDEMVREAKKKGGAFIQNKPSDADEYSFQLAKDDLVVLATDGVTDNISSDDIQLFFRD---NEAMIEKDLQ-------------SVSQQ
TdelPtc7-spliced    186 ANLGDSWCGVFRDSKLVFQTRFQTVGFNAPYQLAIIPDEMVREAKKKGGAFIQNKPSDADEYSFQLAKDDLVVLATDGVTDNISSDDIQLFFRD---NEAMIEKDLQ-------------SVSQQ
CalbPtc7            198 ANLGDSWCGLFRDSKLINETNFQTHNFNTPFQLAKIPEEIVRQAKLQGRRYIIDSPEAADEYTWDLKSGDVVMFATDGVTDNVIPQDIELFLKD-----HEETNQLD-------------DVANK
YlipPtc7            171 ANLGDSGFMVFRNGKLAGGSKAQTHAFNTPYQLAIIPDELKRSDERQGLRHIEDTPAMADQFSFTAEPGDVIVLATDGLTDNMSAQDTLKIVNETMLEHGSWIKDDKEGIKSSGEHKGAMDLARR
consensus           251 aNLGDSwcgvfRdsklvfqtkfQtvgFNaPyQLsiIPd mlkea r g  yIqntP daDeysfql knDivilATDGvtDNiatdDielflkd   n a    elq              ls

ScerPtc7-unspliced  339 FVDNVVSLSKDPNYPSVFAQEISKLTGKNYSGGKEDDITVVVVRVD-
ScerPtc7-spliced    308 FVDNVVSLSKDPNYPSVFAQEISKLTGKNYSGGKEDDITVVVVRVD-
SbayPtc7-unspliced  339 FVKNVVSLSKDPNYPSVFAQEISKLTGKNYSGGKEDDITVVVVRVD-
SbayPtc7-spliced    308 FVKNVVSLSKDPNYPSVFAQEISKLTGKNYSGGKEDDITVVVVRVD-
CglaPtc7-unspliced  331 LVKKVVKISKDPEFPSVFAQEISRLTGKLYKGGKEDDITMVVVKVE-
CglaPtc7-spliced    301 LVKKVVKISKDPEFPSVFAQEISRLTGKLYKGGKEDDITMVVVKVE-
NdaiPtc7-unspliced  350 FVSKVVNLSKDPDYPSVFSQEYSRLTGRLYKGGKEDDITVVLVKVE-
NdaiPtc7-spliced    326 FVSKVVNLSKDPDYPSVFSQEYSRLTGRLYKGGKEDDITVVLVKVE-
NcasPtc7-unspliced  332 FVKKVVALSKDPNYPSLFSQELSKLTGKPYRGGKEDDITVVVVKVE-
NcasPtc7-spliced    313 FVKKVVALSKDPNYPSLFSQELSKLTGKPYRGGKEDDITVVVVKVE-
KafrPtc7-unspliced  332 FVSKVVSLSKDPEYPSVFAQELSKLTGKTYGGGKQDDITVVVVKAM-
KafrPtc7-spliced    307 FVSKVVSLSKDPEYPSVFAQELSKLTGKTYGGGKQDDITVVVVKAM-
KnagPtc7-unspliced  347 LVAKIVTISKDPKFPSVFSQELTKLTGKLYQGGKEDDITMVMVHVK-
KnagPtc7-spliced    318 LVAKIVTISKDPKFPSVFSQELTKLTGKLYQGGKEDDITMVMVHVK-
TphaPtc7-unspliced  341 FVKEVVAISIDPDFPSVFAQEISKLTGKDYRGGKEDDITVVVVKAE-
TphaPtc7-spliced    312 FVKEVVAISIDPDFPSVFAQEISKLTGKDYRGGKEDDITVVVVKAE-
TblaPtc7a           299 LLNYAEKLSKDPNYESVFAKEFTKMSGQYYVGGKEDDITMIYVQVN-
TblaPtc7b           338 LVQNIVKISKDENFPSVFAQEYTNYTGVPCKGGKQDDITMILIRVN-
TdelPtc7-unspliced  333 FVSKVVELSKDPNYPSVFAQEITKLTGKDYRGGKEDDITVVVVKVE-
TdelPtc7-spliced    305 FVSKVVELSKDPNYPSVFAQEITKLTGKDYRGGKEDDITVVVVKVE-
CalbPtc7            315 FVKEVVKVSKDSNFPSAFAQELSRLTGQKYLGGKEDDITVVLVKVK-
YlipPtc7            296 IVLKAKSLSTNKQHLSPFAKEVQQVMKVHYMGGKPDDITVLVVIVNE
consensus           376 fv kvv lSkdpnypSvFaqEiskltgk ykGGKeDDITvvvvkv d
```
