# Supplementary figures and images for: Alternative Splicing and Subfunctionalization Generates Functional Diversity in Fungal Proteomes
Source: PLoS Genet. 2013 Mar 14;9(3):e1003376. doi: 10.1371/journal.pgen.1003376 (PMC3597508; doi:10.1371/journal.pgen.1003376)

## Human Hbs1L

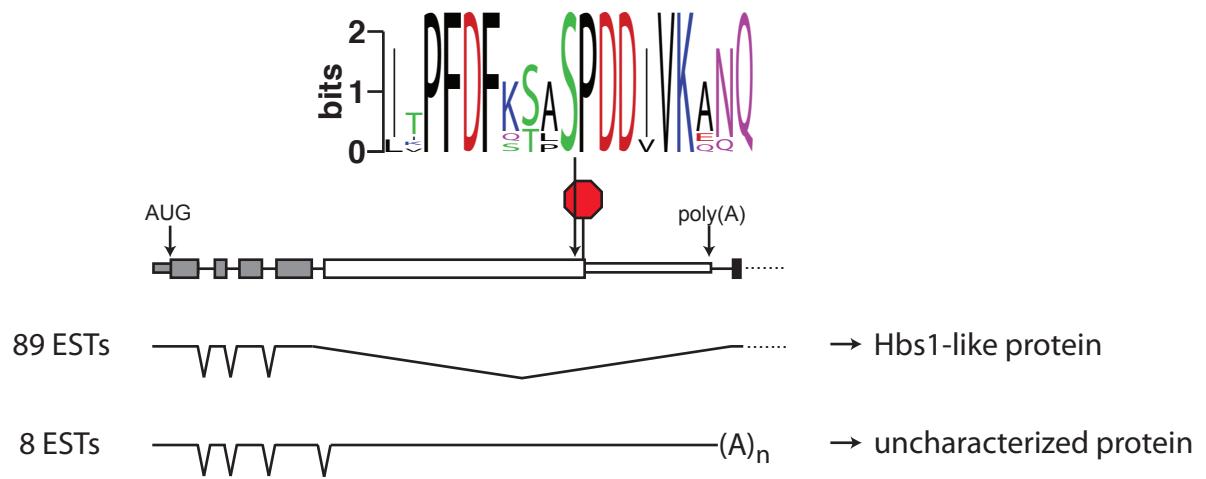

Supplement: Figure S5 — Potential for alternative splicing in the human Hbs1L gene. The gene structure is indicated as in Figure 1, except that only the first 6 exons are shown. A previously annotated HBS1L protein is supported by 89 ESTs and is similar to Hbs1 along it's entire length. To generate this HBS1L the 5th exon is skipped. In addition 8 ESTs suggest that an alternative ORF is generated b including exon 5 as the last exon. This mRNA would encode a truncated protein that includes a conserved sequence motif that is similar to motif S3 of Ski7 described in Figure S1. (PDF) [file pgen.1003376.s005.pdf]
