# Supplementary material for: Alternative Splicing and Subfunctionalization Generates Functional Diversity in Fungal Proteomes
Source: PLoS Genet. 2013 Mar 14;9(3):e1003376. doi: 10.1371/journal.pgen.1003376 (PMC3597508; doi:10.1371/journal.pgen.1003376)
Supplement: Text S1 — Alternative splicing and subfunctionalization of SKI7/HBS1 may also occur in plants and animals. (DOCX) [file pgen.1003376.s006.docx]

Text S1:

**Alternative splicing of the basidiomycete *SKI7/HBS1*genes.**

As discussed in the main text, the *SKI7/HBS1* gene is annotated with 9 exons in *C. neoformans* and 4 exons in *Ustilago maydis*. Existing annotations suggest that a Ski7-like protein can be encoded by inclusion of exons 4 and 5 in *C. neoformans* (or exon 2 in *U. maydis*), but several lines of evidence suggests an alternative where the annotated intron 4 of C. neoformans (and intron 2 of U. maydis) is not a true intron. First, the annotated splice sites are poor matches to the consensus splice sites. Second, neither EST sequences, nor RNA-seq data included in the MIPS Ustilago maydis Database (http://mips.helmholtz-muenchen.de/genre/proj/ustilago/) support splicing of these introns. Instead, one EST suggests that *C. neoformans* intron 4 is not spliced, but that instead exon 4, intron 4 and exon 5 form one large exon. Third, the beginning of *C. neoformans* intron 4 and the corresponding *C. gattii* sequence are perfectly conserved at the amino acid level for 38 codons, ending in a stop codon. Fourth, this conserved amino acid sequence is similar to sequence motif S3 described above for ascomycetes and to the protein sequence in U. maydis. Thus in addition to the existing annotation, a truncated Ski7-like protein may be generated that terminates at a stop codon in what is annotated as intron 4 in *C. neoformans* and intron 2 in *U. maydis*.

**Metazoan *SKI7/HBS1* homologs share characteristics with both Ski7 and Hbs1, suggesting that both Ski7 and Hbs1 functions are ancient.**

It has been suggested that Ski7 function is a relatively recent invention that arose post WGD. In the main manuscript we show that this is not true, but that instead Ski7 function was present in the common ancestor of the *Ascomycota* and *Basidiomycota*. Sequence analysis of animal and plant homologs suggest that both functions may be even older.

A human homolog of Hbs1 has been described and named Hbs1L. Hbs1L encodes a protein of 71 KDa or 75 KDa, depending on whether intron 3 is included or excluded. These two isoforms closely resemble fungal Hbs1 in both sequence and in *in vitro* biochemical activity, consistent with their proposed role in no-go mRNA decay ([1-3](#_ENREF_1)). In addition to these two splice forms, there appears to be a third splice form that has not been studied. This third isoform is of similar size (70 KDa), but only the first 144 amino acids, encoded by exons 1 through 4, are in common. In splice forms 1 and 2, exon 4 is spliced to exons 6 through 19 (supplemental figure 5). In the third splice form, exon 5 is included, and the mRNA ends with a cleavage and polyadenylation site at the end of exon 5. Several observations suggest that this third isoform is a biologically relevant distinct protein that shares some characteristics with Ski7. Proteins similar to this third isoform have been annotated in a variety of animals, including several mammals, *Xenopus*, and sea anemone. In other eukaryotes, a similar protein has not been annotated, but in some cases, the coding potential of exon 5 is conserved (*e.g.* zebrafish and chicken). This conservation within animals support that this is a functional isoform. One of the most highly conserved sequence motifs in splice form 3 is PFDFxxxSPDDIVKxNQ (supplemental figure 5), which resembles the Ski7 specific motif 3 described in the main text. Strikingly, splice form 3 resembles the possible truncated basidiomycete proteins described in the main text in that it motif S3 is near the C-terminus and GTPase domains are not present. The GTPase domains of *S. cerevisiae* Ski7 are required for the recognition of nonstop mRNA, but a mutant version lacking these domains is fully functional for exosome-mediated decay of other mRNAs ([4](#_ENREF_4), [5](#_ENREF_5)). This suggests that splice form three of Hbs1L (and the basidiomycete proteins) may be an exosome cofactor, but may not be responsible for recognition of nonstop mRNAs. These observations further suggest that alternative splicing of SKI7/HBS1 was already present in the common ancestor of animals and fungi.

**Duplication and possible subfunctionalization of SKI7/HBS1 genes in plants.**

Plant homologs to Hbs1 have not been previously studied, but we detected a single homolog in the Arabidopsis genome (AT5G10630) and two homologs in rice (Os01g0116600 and Os04g0677800). The Arabidopsis homolog and one of the rice homologs contain motifs similar to the Ski7-specific motif 3 (PFKFDAPSPDDLV and PFKFDTPSPDDVA, respectively), but the second rice gene lacks a similar motif. Thus, we speculate that the *SKI7/HBS1* gene may have been duplicated and subfunctionalized in the rice lineage, similar to the *Saccharomyces* and *Schizosaccharomyces* lineages.

([6-8](#_ENREF_6))

([7](#_ENREF_7))

1. Shoemaker CJ, Eyler DE, & Green R (2010) Dom34:Hbs1 promotes subunit dissociation and peptidyl-tRNA drop-off to initiate no-go decay. *Science* 330(6002):369-372.

2. Pisareva VP, Skabkin MA, Hellen CU, Pestova TV, & Pisarev AV (2011) Dissociation by Pelota, Hbs1 and ABCE1 of mammalian vacant 80S ribosomes and stalled elongation complexes. *The EMBO journal* 30(9):1804-1817.

3. Shoemaker CJ & Green R (2011) Kinetic analysis reveals the ordered coupling of translation termination and ribosome recycling in yeast. *Proceedings of the National Academy of Sciences of the United States of America* 108(51):E1392-1398.

4. van Hoof A, Frischmeyer PA, Dietz HC, & Parker R (2002) Exosome-mediated recognition and degradation of mRNAs lacking a termination codon. *Science* 295(5563):2262-2264.

5. Meaux S & van Hoof A (2006) Yeast transcripts cleaved by an internal ribozyme provide new insight into the role of the cap and poly(A) tail in translation and mRNA decay. *RNA* 12(7):1323-1337.

6. Becker T*, et al.* (2011) Structure of the no-go mRNA decay complex Dom34-Hbs1 bound to a stalled 80S ribosome. *Nature structural & molecular biology* 18(6):715-720.

7. Chen L*, et al.* (2010) Structure of the Dom34-Hbs1 complex and implications for no-go decay. *Nature structural & molecular biology* 17(10):1233-1240.

8. van den Elzen AM*, et al.* (2010) Dissection of Dom34-Hbs1 reveals independent functions in two RNA quality control pathways. *Nature structural & molecular biology* 17(12):1446-1452.
